# Prestroke physical activity and outcomes after intracerebral haemorrhage in comparison to ischaemic stroke: protocol for a matched cohort study (part of PAPSIGOT)

**DOI:** 10.1136/bmjopen-2021-053067

**Published:** 2021-11-19

**Authors:** Adam Viktorisson, Dongni Buvarp, Katharina S Sunnerhagen

**Affiliations:** Inst of Neuroscience and Physiology, Univ of Gothenburg, Gothenburg, Sweden

**Keywords:** statistics & research methods, stroke medicine, protocols & guidelines, preventive medicine

## Abstract

**Introduction:**

Piling evidence suggests that a higher level of prestroke physical activity can decrease stroke severity, and reduce the risk of poststroke mortality. However, prior studies have only included ischaemic stroke cases, or a majority of such. We aim to investigate how premorbid physical activity influences admission stroke severity and poststroke mortality in patients with intracerebral haemorrhage, compared with ischaemic stroke. A prespecified analysis plan counteract some inherent biases in observational studies, and promotes transparency.

**Methods and analysis:**

This is a statistical analysis protocol for a matched cohort study, including all adult patients with intracerebral haemorrhage, and matched ischaemic stroke controls, treated at Sahlgrenska University Hospital in Sweden between 1 November 2014 and 30 June 2019. All patients have been identified in the Väststroke register, and the data file has been sent for merging with national registries. The follow-up of time for survival will be approximately 2–7 years. The sample size calculation indicates that a minimum of 628 patients with intracerebral haemorrhage is needed for power of 80% at an alpha level of 0.01. Multiple imputation by chained equations will be used to handle missing data. The entire cohort of patients with intracerebral haemorrhage will be matched with consecutive ischaemic stroke controls (1:3 ratio) using nearest neighbour propensity score matching. The association between prestroke physical activity and admission stroke severity will be evaluated using multivariable ordinal regression models, and risk for all-cause mortality will be analysed using multivariable Cox proportional-hazards models. Potential confounders include age, ethnicity, income, educational level, comorbidity, medical treatments, alcohol-related disorders, drug abuse and smoking.

**Ethics:**

Data collection for the Physical Activity Pre-Stroke In GOThenburg project was approved by the Regional Ethical Board on 4 May 2016. An additional application was approved by the National Ethical Review Authority on 7 July 2021.

**Strengths and limitations of this study**

- A prespecified sample size calculation and statistical analysis plan decrease bias in observational research.
- The novel relationship between prestroke physical activity and outcomes after intracerebral haemorrhage will be studied in a large sample.
- Comprehensive covariate adjustment will enhance the reliability of the results.
- The retrospective analysis of registry data is a limitation of the study.
- Self-reported assessments of prestroke physical activity introduce recall bias, which is another limitation of the study.

## Introduction

Observational studies are of great value in cases when a randomised controlled trial would be unethical, impractical or untimely. Observational studies are, however, subjected to several types of selection and reporting bias, which can lead to a lack in reproducibility. The reliability of observational data can be enhanced by emulating principal design aspects of randomised trials.^1^ Prespecified eligibility criteria, a priori sample size estimation, definition of exposure and outcome measures, a standardised follow-up period, and a statistical analysis plan may counterbalance some inherent limitations of the observational study design and promote transparency.^2^

Physical activity is an example of exposure that is hard to study in a randomised, controlled setting. The relationship between premorbid physical activity and admission stroke severity, as well as poststroke mortality has been analysed in several observational studies, with varying results. The majority of prior studies have found that prestroke physical activity reduce stroke severity,^3–8^ in-hospital mortality,^8^ cardiovascular mortality^9^ and all-cause mortality.^9–12^ However, some studies found no associations between physical activity and stroke severity,^13 14^ or poststroke mortality after covariate adjustment.^15 16^ These studies differ in data collection strategies, sample sizes, physical activity assessments and statistical analyses. Moreover, none of the prior studies have investigated the association between prestroke physical activity and stroke severity or mortality in the subgroup of patients with intracerebral haemorrhage (ICH).

Here, we present a statistical analysis plan, and give the rationale for an observational, matched cohort study part of the Physical Activity Pre-Stroke In GOThenburg (PAPSIGOT) project. We also provide a narrative discussion for potential confounding factors to the effect of prestroke physical activity on outcomes after ICH. This study protocol follows the guidelines for reporting of statistical analysis plans in clinical trials, published in the Journal of the American Medical Association.^17^

### Research objectives

(1) To investigate the relationship between prestroke physical activity and admission stroke severity in patients with ICH, compared with patients with ischaemic stroke. (2) To investigate how prestroke physical activity influence the risk of mortality after ICH, in comparison to ischaemic stroke.

## Methods

This is a statistical analysis protocol for a matched cohort study. Prestroke physical activity is the main independent variable. Admission stroke severity and all-cause mortality are the outcome variables of interest. All adult patients with ICH and ischaemic stroke, treated at Sahlgrenska University Hospital between 1 November 2014 and 30 June 2019 have been identified in the Väststroke register. The comprehensive stroke units at Sahlgrenska University Hospital has a catchment area of 700 000 people, and patients who had a stroke in nearby regions requiring thrombectomy or neurosurgical monitoring are transported to this hospital. All persons with stroke in Sweden have equal access to tax-funded healthcare services, which ensures the representativeness of this sample.

### Data collection and management

Prestroke physical activity and acute stroke severity assessments were collected from the Väststroke register, and cross-references with medical records to minimise missing data. Physical activity was assessed using the four-level Saltin-Grimby Physical Activity Level Scale (SGPALS).^18 19^ Acute stroke severity was assessed using the National Institutes of Health Stroke Scale (NIHSS).^20^ The data collected from the Väststroke register has been sent for merging with national registries. Information regarding prestroke conditions and treatments will be collected from the Swedish National Stroke Registry (Riksstroke). Country of birth will be collected from the Swedish Multi-Generation register, and socioeconomic variables will be collected from the Longitudinal Integration Database for Health Insurance and Labour Market Studies held by Statistics Sweden. Comorbidities will be collected from the National Patient Registry, and mortality rates will be collected from the Swedish Cause of Death registry, held by the National Board of Health and Welfare. All-cause mortality and cerebrovascular mortality will be recorded for all patients until October 2021, which allow for a minimum follow-up of 2 years.

The data management started on 17 May 2021 after submission of this protocol. The final data was recived on 19 October 2021. Patients without a Swedish personal identification number, and patients with non-specified aetiology will be excluded. The final sample is will consist of 763 patients with ICH.

Continuous variables will be either mean-centred or dichotomised when appropriate. For ordinal variables, categories with limited observations will be merged on the base of clinical reasoning. All variables and data sources are listed in table 1. The categories of SGPALS will be collapsed into inactive (SGPALS level 1) and physically active (SGPALS levels 2–4) for all analyses. NIHSS scores will be categorised based on distributional assumptions.^6^ The weighted Charlson Comorbidity Index will be calculated, and applied as a four-level ordinal variable using the ICD-10 diagnoses specified in table 1. The 1-year mortality risk described by Charlson *et al* was 12% for patients with 0 points, 26% for 1–2 points, 52% for 3–4 points and 85% for ≥5 points.^21^

**Table 1:** Registers and variables included in the study

The väststroke register	
Age	Mean centred or dichotomised.
Sex	Female/male.
Physical activity assessment (SGPALS)	Mostly sedentary (level 1); light physical activity such as walking ≥4 hours/week (level 2); moderate physical activity such as running ≥2 hours/week (level 3); hard physical training for competition sports several times per week (level 4).
Stroke severity assessment (NIHSS)	Mild stroke (NIHSS 0-5), moderate stroke (NIHSS 6-14), and severe stroke (NIHSS >14).
**The Riksstroke register**	
Prior stroke	Yes/no.
Prior transient ischaemic attack	Yes/no.
Atrial fibrillation	Yes/no.
Diabetes	Yes/no.
Regular smoking the year before stroke	Yes/no.
Antihypertensive treatment	Yes/no.
Statin treatment	Yes/no.
Antiplatelet treatment	Yes/no.
Anticoagulant treatment	Yes/no.
**The LISA register**	
Country of birth	Born in Sweden/born outside of Sweden.
Income	Mean centred or dichotomised.
Educational level	Primary school (<10 years), secondary school (10–12 years) and postsecondary or university education (>12 years).
**The NPR**	
Charlson Comorbidity Index: myocardial infarction, heart failure, peripheral vascular disease, dementia, pulmonary disease, connective tissue disease, peptic ulcer disease liver disease, diabetes, Hemiplegia or paraplegia, renal disease, cancer, HIV/AIDS	Low (0 points), moderate (1–2 points), high (3–4 points) and very high (≥5 points) risk. ICD-10 codes: I21, I22, I25.2, I42, I11.0, I13.0, I13.2 I50, I25.5, I65, I70-I74, I77, I73.9, I79.0, R02, F00-F03, F05.1, J40-J47, J60-J67, M05.0-M05.3, M05.8-M06.0, M06.3, M06.9, M32, M33.2, M34, M35.3, K25-K28, K70.2, K70.3, K71.7, K73, k74.0, K74.2-K74.6, K72.9, K76.6, K76.7, K72.1, E10.1, E10.5, E10.9, E11.1, E11.5, E11.9, E13.1, E13.5, E13.9, E14.1, E14.5, E14.9, E10.2-E10.4, E11.2-E11.4, E13.2-E13.4, E14.2-E14.4, G80.0, G80.2, G81, G82, N01-N08, N11, N14-N19, N25, C00-C76, C90, C97, C91-C95, C81-C86, C88, C77-C80, B20-B24
Depression	Yes/no. ICD-10 codes: F32-F33
Psychotic diseases	Yes/no. ICD-10 codes: F20-F29
Alcohol related disorders	Yes/no. ICD-10 codes: F10, K70.1-K70.4, K70.9, K74.0
Drug abuse	Yes/no. ICD-10 codes: F11, T40.0-T40.4, F12, T40.7, F14, T40.5, F15, T43.6, F16, T40.6, F19
**The Swedish cause of death register**	ICD-10 code
All-cause mortality	–
Cerebrovascular mortality	ICD-10 codes: I60-I69

ICD-10, International Classification of Diseases, Tenth Revision; LISA, Longitudinal Integration Database for Health Insurance and Labour Market Studies; NIHSS, National Institutes of Health Stroke Scale; NPR, National Patient Registry; SGPALS, Saltin-Grimby Physical Activity Level Scale.

### Sample size calculation

The sample size was estimated using the formula for ordered categorical data suggested by John Whitehead, stated below.^22^ The primary outcome measure for the power calculation is the difference in stroke severity on a three-level scale between inactive and physically active patients. Control probabilities were collected from previous research (table 2).^6^ We expect a fraction of 60% physically inactive patients (SGPALS level 1). The sample size estimate was calculated to detect an OR of 2.0 at an alpha level of 0.01 with a power of 80.^6^ Based on the power calculation, we need a minimum of 628 patients with ICH, which is within the expected number of cases.

**Table 2:** Frequencies of stroke severity using the National Institutes of Health Stroke Scale (NIHSS), in physically inactive and active patients with stroke (Reinholdsson *et al*)^6^

	No physical activity $\pi_{C}$	Physical activity $\pi_{E}$	Cumulative OR
Mild stroke (NIHSS 0–5)	73.6%	86.5%	0.44
Moderate stroke (NIHSS 6–14)	20.4%	10.8%	0.43
Severe stroke (NIHSS >14)	6.0%	2.7%	-

$$n=6{\left(Z_{1-\frac{\alpha }{2}}+Z_{\beta }\right)}^{2}/\left(log{\left(OR\right)}^{2}\left[1-\sum_{i=1}^{k}{\left(\left(\pi_{iE}+\pi_{iC}\right)/2\right)}^{3}\right]\right)$$

### Statistical analyses

Data will be analysed using R, V.4.0.2 (R Core Team. R: A language and environment for statistical computing. R Foundation for Statistical Computing, Vienna, Austria.), and SPSS Statistics for Windows, V.27.0 (released on 2018; IBM). All statistical testing will be interpreted at a two-tailed significance level of 0.01 to adjust for multiple comparisons.

### Missing data

Missing observations for each variable will be explored, and variables with more than 30% will be excluded from the data set. Individuals with missing values on more than 20% of the remaining variables will be excluded from analyses. The number of missing observations in the total data set is expected to be below 3%. Multiple imputation by chained equations will be applied to handle missing data. We will use the predictive mean matching method, originally proposed by Rubin,^23^ implemented for multiple imputation.^24^ The method generates a separate model for each variable with missing data, and creates multiple imputations for each value. The predictive mean algorithm preserves the original data distribution and allows for imputation of continuous, binary and categorical data.^25^ The imputation will be performed separately for patients with ICH and ischaemic stroke, and ordered from the variable with least missing values (monotone). Only variables with a minimum correlation of 0.1 and at least 50% data will be used for prediction. The number of multiple imputations will be set to m=20.

### Propensity scores and matching

To achieve equipoise between patients with ICH and ischaemic stroke, propensity score matching will be applied. We will include the entire cohort of patients with ICH and match them with consecutive ischaemic stroke patients, in a 1:3 ratio. The propensity score will be based on sex and potential confounding variables. The rational for the selection of confounders is given below. Here, we assume that confounders for the effect of physical activity on stroke severity will be analogous for ICH and ischaemic stroke. We will conduct generalised boosted regression modelling to calculate the propensity scores.^26^ We intend to estimate the average eﬀect of being physically active, that is, the average treatment effect for the treated will be applied. The number of trees, shrinkage and interaction depth will be tuned by using 10-fold cross-validation. We will use the stopping rule es.mean, which uses the absolute standardised bias as balance metric and the mean of the covariate balance metrics to summarise across variables. The propensity score matching will be conducted using the nearest neighbour matching method, with a logistic distance measure and without replacement.^27^

### Regression models

We will conduct separate models for haemorrhagic and ischaemic stroke cases, respectively. All models will be adjusted for potential confounders. The association between prestroke physical activity and admission stroke severity will be evaluated using multivariable ordinal regression models.^28^ For the ordinal regression, NIHSS scores will be trichotomised. A subgroup analysis will be performed for patients with first-time ICH. Prior to the analyses, absence of multicollinearity, and the proportional odds assumption will be checked. Diagnostics will be performed using surrogate residuals.^29^

The association between prestroke physical activity and all-cause mortality will be evaluated using multivariable Cox proportional-hazards models.^30^ Additional models will be fitted adjusted for admission stroke severity.^31^ Model diagnostics will be applied to evaluate violations of the proportional hazards assumption, random censoring and functional forms of continuous covariates.^32^ Furthermore, outlying and influential observations will be checked for all models.

### Patient and public involvement

There were no patients or members of the public involved in the planning of this study.

### Confounding variables

**Figure 1:**
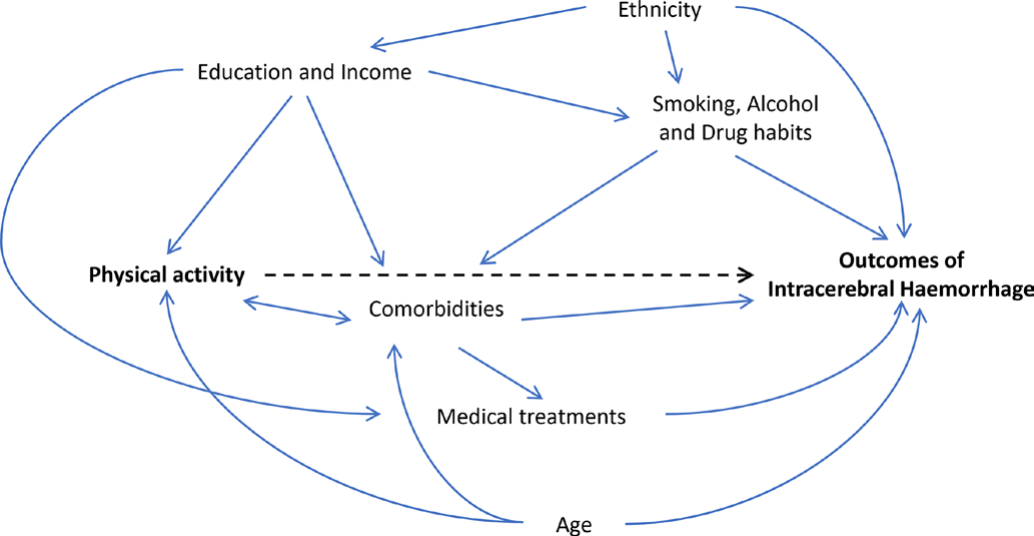
Directed acyclic graph for confounders to the effect of physical activity on outcomes after intracerebral haemorrhage (stroke severity and poststroke mortality). Solid lines indicate the relationships between covariates, and the dotted line indicate the causal effect of physical activity.

There is limited evidence for the relationship between physical activity and previously known determinants of outcomes following ICH. However, several factors may confound or mediate the potential impact of physical activity. The covariates included as potential confounders in this study are based on theoretical reasoning, and current evidence. The relationship between prestroke physical activity and the main groups of confounders is portrayed in figure 1.

Degenerative vascular changes are frequent in the ageing population. In addition to being a risk factor for all stroke, higher age has been identified as an important predictor of disability, and decreased long-term survival in ICH^33 34^ A person’s age also influences the probability of being physically active,^35^ and should therefore be considered a confounding factor. Sex, however, did not significantly influence the severity of haemorrhagic stroke in a meta-analysis of eight population‐based stroke incidence studies.^36^

Socioeconomic status-related differences influence haemorrhagic stroke severity, but could partly be explained by differences in comorbidities, disparity in health and care services, ethnicity, stress, job strain and life style factors. Income, education and living alone have been associated with mortality following the acute phase of stroke.^37^ Low income was found to increase the absolute risk of death at 3 months after ICH, and the effect was largely mediated by initial stroke severity.^38^ In adulthood, socioeconomic variables are associated with the propensity of being physically active.^39^ Among young people, a recent study found that haemorrhagic stroke survivors of African and Hispanic ethnicity have better outcomes compared with those of Caucasian descent.^40^ In the current study, country of birth will be used as a surrogate measure for ethnicity.

Active cigarette smoking has been associated with increased stroke severity,^41 42^ and premorbid alcohol consumption has been identified as independent determinant of functional status and mortality after ICH.^43^ Cigarette smoking and physical activity are intuitively incongruent behaviours, and both positive and negative associations has been described.^44^ Likewise, alcohol consumption has in some studies been found to be a contributor to sedentary behaviour.^45^

Patients with severe comorbidity may experience worse outcomes following ICH, although this has not been thoroughly investigated. One study found that dialysis patients with a medical history of stroke, diabetes and malignancy had a higher risk of mortality following ICH.^46^ In another study, global comorbidity evaluated by the Charlson Comorbidity Index was associated with increased risk of 90-day mortality, but not with the length of hospital stay, among immobile patients below 50 years of age.^47^ Severe disease may limit a person’s ability to be physically active, and thereby confound the effect of prestroke physical activity on outcomes following ICH.

Psychiatric comorbidity has been associated with stroke severity and functional outcomes in a population with both haemorrhagic and ischaemic stroke cases.^48^ Physical activity decrease the risk of depression, and a majority of people with depression report sedentary behaviour.^49 50^ Similarly, patients with psychotic diseases, such as Schizophrenia, generally have a very low level of physical activity.^51^

Hyperglycaemia and diabetes have been related to worse outcomes after ICH. Diabetes and high blood glucose in nondiabetic patients have been associated with cerebral complications, and are independent predictors of 30-day mortality, and 3-month mortality following ICH.^52^ Hyperglycaemia has also been found to decrease short term survival after ICH.^53 54^ The presence of hyperglycaemia in severe stroke may, however, be a result of an extensive cerebral lesion, causing a phycological increment of cortisol and catecholamines. Regular physical activity promotes blood glucose control in diabetic patients.^55^ Still, a large survey of the US population found that diabetic adults generally engaged in less physical activities compared with non-diabetic adults.

Atrial fibrillation is present in about one third of patients 3 months after ICH,^56^ and premorbid atrial fibrillation has been found to predict a higher risk of death 3 months after ictus.^57^ However, the risk increment seemed to be mediated by the higher prevalence of antithrombotic treatment in the group with atrial fibrillation. There is a complicated relationship between physical activity and the risk of atrial fibrillation. While a moderate amount of physical activity is protective for atrial fibrillation, vigorous physical activity increase the risk of atrial fibrillation in men.^58^

Coagulopathy caused by anticoagulants increase the risk of haematoma expansion, mortality and disability after ICH. Warfarin usage has been associated with increased risk of in-hospital haematoma expansion,^59^ and a doubled risk of mortality at 3 months after ICH.^60^ In an international multicentre pooled analysis, haemorrhagic volume on admission, haematoma expansion, functional status at discharge and 90-day mortality were similar in patients treated with warfarin and non-vitamin K antagonist oral anticoagulation (NOAK), meaning that the risk increment is substantial for treatment with NOAK as well.^61^ The propensity of being physically active may be connected to anticoagulant treatment through differences in the prevalence of atrial fibrillation and previous strokes.

The use of lipid-lowering drugs has been controversial in patients with ICH as hypocholesterolaemia has been identified as a risk factor for ICH and haematoma expansion.^62 63^ Low levels of serum lipids were also independently associated with a poor 3 months prognosis after ICH in women.^64^ The use of statins has, however, only been correlated to an increased risk of ICH in patients with a prior haemorrhagic or lacunar ischaemic stroke. On the other hand, several observational studies have reported better outcomes after ICH among patients medicated with statins. Premorbid statin usage was associated with a better long-term outcome at 12 months after ICH.^65^ Another study found that statins reduced mortality and disability without any negative effect on haematoma growth, but the association disappeared when adjusted for potential indication bias.^66^ Regular physical activity positively alter the levels of blood lipids, and may thereby decrease the probability of receiving statin treatment.^67^

A previous stroke or transient ischaemic attack (TIA) can potentially precondition the brain to endure ischaemia, and having gone through a TIA has been associated with lower ischaemic stroke severity.^3^ Although ischaemic tolerance may play an important role in haemorrhagic stroke severity, the influence of a preceding stroke or TIA has not yet been evaluated. Patients diagnosed with stroke or TIA are, however, likely to receive recommendations regarding physical activity in accordance poststroke guidelines.

In conclusion, the following potential confounders will be collected, and adjusted for: age, ethnicity, income, educational level, comorbidity, medical treatments, alcohol related disorders, drug abuse and smoking. The comorbidities accounted for are prior TIA, prior stroke, diabetes, psychiatric disorders and Charlson Comorbidity Index. The medical treatments accounted for are anticoagulant treatment, antihypertensive treatment, antiplatelet treatment and statin treatment.

### Ethics

Data collection for the PAPSIGOT project was approved by the Regional Ethical Board of Gothenburg on 4 May 2016 (346-16). An additional application was approved by the Swedish Ethical Review Authority on 7 July 2021 (2021-03324). Research within the framework of quality registers does not require written informed consent from included patients. The Personal Data Act (Swedish law #1998:204, issued 29 April 1998) allows data from medical records to be collected for quality control without receiving a written informed consent.

## Supplementary Material

Author's manuscript
