# Supplementary material for: Prestroke physical activity and outcomes after intracerebral haemorrhage in comparison to ischaemic stroke: protocol for a matched cohort study (part of PAPSIGOT)
Source: BMJ Open. 2021 Nov 19;11(11):e053067. doi: 10.1136/bmjopen-2021-053067 (PMC8606775; doi:10.1136/bmjopen-2021-053067)
Supplement: Author's manuscript [file bmjopen-2021-053067.draft_revisions.pdf]

BMJ Open is committed to open peer review. As part of this commitment we make the peer review history of every article we publish publicly available.

When an article is published we post the peer reviewers' comments and the authors' responses online. We also post the versions of the paper that were used during peer review. These are the versions that the peer review comments apply to.

The versions of the paper that follow are the versions that were submitted during the peer review process. They are not the versions of record or the final published versions. They should not be cited or distributed as the published version of this manuscript.

BMJ Open is an open access journal and the full, final, typeset and author-corrected version of record of the manuscript is available on our site with no access controls, subscription charges or pay-per-view fees (http://bmjopen.bmj.com).

If you have any questions on BMJ Open's open peer review process please email info.bmjopen@bmj.com

BMJ Open

# Pre-stroke physical activity and outcomes after primary intracerebral haemorrhage: protocol for a matched cohort study (part of PAPSIGOT)

| | |
|---|---|
| Journal: | *BMJ Open* |
| Manuscript ID | bmjopen-2021-053067 |
| Article Type: | Protocol |
| Date Submitted by the Author: | 03-May-2021 |
| Complete List of Authors: | Viktorisson, Adam; Goteborgs universitet Institutionen for neurovetenskap och fysiologi, Rehabilitation medicine<br>Buvarp, Dongni; University of Gothenburg<br>Sunnerhagen, Katharina; Univ of Gothenburg, Inst of Neuroscience and Physiology |
| Keywords: | STATISTICS & RESEARCH METHODS, STROKE MEDICINE, Protocols & guidelines < HEALTH SERVICES ADMINISTRATION & MANAGEMENT, PREVENTIVE MEDICINE |
| | |

SCHOLARONE™
Manuscripts

*I, the Submitting Author has the right to grant and does grant on behalf of all authors of the Work (as defined in the below author licence), an exclusive licence and/or a non-exclusive licence for contributions from authors who are: i) UK Crown employees; ii) where BMJ has agreed a CC-BY licence shall apply, and/or iii) in accordance with the terms applicable for US Federal Government officers or employees acting as part of their official duties; on a worldwide, perpetual, irrevocable, royalty-free basis to BMJ Publishing Group Ltd ("BMJ") its licensees and where the relevant Journal is co-owned by BMJ to the co-owners of the Journal, to publish the Work in this journal and any other BMJ products and to exploit all rights, as set out in our licence.*

*The Submitting Author accepts and understands that any supply made under these terms is made by BMJ to the Submitting Author unless you are acting as an employee on behalf of your employer or a postgraduate student of an affiliated institution which is paying any applicable article publishing charge ("APC") for Open Access articles. Where the Submitting Author wishes to make the Work available on an Open Access basis (and intends to pay the relevant APC), the terms of reuse of such Open Access shall be governed by a Creative Commons licence – details of these licences and which Creative Commons licence will apply to this Work are set out in our licence referred to above.*

*Other than as permitted in any relevant BMJ Author's Self Archiving Policies, I confirm this Work has not been accepted for publication elsewhere, is not being considered for publication elsewhere and does not duplicate material already published. I confirm all authors consent to publication of this Work and authorise the granting of this licence.*

For peer review only

# Pre-stroke physical activity and outcomes after primary intracerebral haemorrhage: protocol for a matched cohort study (part of PAPSIGOT)

Adam Viktorisson (MD) [1,*], Dongni Buvarp (MD, PhD) [1], Katharina S Sunnerhagen (MD, PhD) [1]

1. Institute of Neuroscience and Physiology, Rehabilitation Medicine, Sahlgrenska Academy, University of Gothenburg, and the Sahlgrenska University Hospital, Gothenburg, Sweden.
2. Institute of Neuroscience and Physiology, Department of Health and Rehabilitation, Sahlgrenska Academy, University of Gothenburg.

**Corresponding author:** Adam Viktorisson
Sahlgrenska Academy, Institute of Neuroscience and Physiology,
University of Gothenburg
Per Dubbsgatan 14, 3rd floor
PO Box 430
SE 405 30, Gothenburg, Sweden.
E-mail address: adam.viktorisson@gu.se
Phone: +46-31-342 2803

**Principal investigator:** Prof. Katharina Stibrant Sunnerhagen
Sahlgrenska Academy, Institute of Neuroscience and Physiology,
University of Gothenburg
E-mail address: ks.sunnerhagen@neuro.gu.se

**SAP version number and date:** version 1.0 2021-05-02

## Abstract

*Introduction*

Piling evidence suggests that a higher level of pre-stroke physical activity can decrease admission stroke severity, and reduce the risk of post-stroke mortality. However, prior studies have only included ischemic stroke cases, or a majority of such. We aim to investigate how premorbid physical activity affect admission stroke severity and post stroke mortality in patients with primary intracerebral haemorrhage. A prespecified analysis plan counteract some of the inherent biases in observational studies, and promotes transparency.

For peer review only

*Methods and analysis*

This is a statistical analysis protocol for a matched cohort study, including all adult patients with primary intracerebral haemorrhage, and matched ischemic stroke controls, treated at Sahlgrenska University Hospital in Sweden between November 1, 2014 and June 30, 2019. All patients have been identified in the Väststroke register, and the data file has been sent for merging with national registries. The follow-up of time for survival will be approximately 2 to 7 years. The sample size calculation indicates that a minimum of 628 patients with intracerebral haemorrhage is needed for power of 80% at an alpha level of 0.01. Multiple imputation by chained equations will be used to handle missing data. The entire cohort of patients with primary intracerebral haemorrhage will be matched with consecutive ischemic stroke controls (1:2 ratio) using nearest neighbour propensity score matching. The association between pre-stroke physical activity and admission stroke severity will be evaluated using multivariable ordinal regression models, and risk for all-cause mortality will be analysed using multivariable Cox Proportional-Hazards models. Potential confounders include age, ethnicity, income, educational level, comorbidity, medical treatments, alcohol related disorders, drug abuse, and smoking.

*Ethics*

Data collection for the Physical Activity Pre-Stroke In GOThenburg (PAPSIGOT) project was approved by the Regional Ethical Board of Gothenburg on May 4, 2016 (346–16), and by the Swedish Ethical Review Authority (amendment 2021-01109).

**Strengths and limitations of this study**

- A prespecified sample size calculation and statistical analysis plan decrease bias in observational research.
- The novel relationship between pre-stroke physical activity and outcomes after primary intracerebral haemorrhage will be studied in a large sample.
- Comprehensive covariate adjustment will enhance the reliability of the results.
- The retrospective analysis of registry data is a limitation of the study.
- Self-reported assessments of pre-stroke physical activity introduce recall bias, which is another limitation of the study.

For peer review only

## Introduction

Observational studies are of great value in cases when a randomized controlled trial would be unethical, impractical or untimely. Observational studies are, however, subjected to several types of selection and reporting bias, which can lead to a lack in reproducibility. The reliability of observational data can be enhanced by emulating principal design aspects of randomized trials.[1] Pre-specified eligibility criteria, a priori sample size estimation, definition of exposure and outcome measures, a standardised follow-up period, and a statistical analysis plan may counterbalance some inherent limitations of the observational study design, and promote transparency.[2]

Regular physical activity is an example of exposures that are hard to study in a randomized, controlled setting. The relationship between premorbid physical activity and admission stroke severity, as well as post-stroke mortality has been analysed in several observational studies, with varying results. The majority of prior studies have found that pre-stroke physical activity reduce stroke severity,[3-8] in-hospital mortality,[8] cardiovascular mortality,[9] and all-cause mortality.[9-12] However, some studies found no associations between physical activity and stroke severity,[13,14] or post-stroke mortality after covariate adjustment.[15,16] These studies differ in data collection strategies, sample sizes, physical activity assessments, and statistical analyses. Moreover, none of the prior studies have investigated the association between pre-stroke physical activity and stroke severity or mortality in the subgroup of patients with intracerebral haemorrhage.

Primary intracerebral haemorrhage is the deadliest type of stroke, and a large proportion of survivors experience severe disability and poor quality of life.[17,18] Today, there a few modifiable factors, and no largely applicable intervention that can improve outcomes for those affected.[19] The global socioeconomic burden is significant, and the incidence of intracerebral haemorrhage is particularly high in low income populations due to a higher prevalence of cerebrovascular risk factors.[20] Further knowledge regarding cost-efficient prevention, and implementation of such, is therefore needed.

Here, we present a statistical analysis plan, and give the rational for an observational, matched cohort study part of the Physical Activity Pre-Stroke In GOThenburg (PAPSIGOT) project. We also provide a narrative discussion for potential confounding factors to the effect of pre-stroke physical activity on outcomes after intracerebral haemorrhage. This study protocol follows the guidelines for reporting of statistical analysis plans in clinical trials, published in the Journal of the American Medical Association.[21]

For peer review only

## Research objectives

(1) To investigate the relationship between pre-stroke physical activity and admission stroke severity in patients with primary intracerebral haemorrhage, compared to patients with ischemic stroke. (2) To investigate how pre-stroke physical activity influence the risk of mortality after primary intracerebral haemorrhage, in comparison to ischemic stroke.

## Methods

This is a statistical analysis protocol for a matched cohort study. All adult patients with primary intracerebral haemorrhage, and ischaemic stroke, treated at Sahlgrenska University Hospital between November 1, 2014 and June 30, 2019 have been identified in the Väststroke register. The comprehensive stroke units at Sahlgrenska University Hospital has a catchment area of 700,000 people, and stroke patients in nearby regions requiring thrombectomy or neurosurgical monitoring are transported to this hospital. All persons with stroke in Sweden have equal access to tax-funded healthcare services, which ensures the representativeness of this sample.

*Data collection and management*

Pre-stroke physical activity and acute stroke severity assessments were collected from the Väststroke register, and cross-references with medical records to minimize missing data. Physical activity was assessed using the four-level Saltin-Grimby Physical Activity Level Scale (SGPALS).[22,23] Acute stroke severity was assessed using the National Institutes of Health Stroke Scale (NIHSS).[24] The data collected from the Väststroke register has been sent for merging with national registries. Information regarding pre-stroke conditions and treatments will be collected from the Swedish National Stroke Registry (Riksstroke). Country of birth will be collected from the Swedish Multi-Generation register, and socioeconomic variables will be collected from the Longitudinal Integration Database for Health Insurance and Labour Market Studies (LISA) held by Statistics Sweden. Comorbidities will be collected from the National Patient Registry (NPR), and mortality rates will be collected from the Swedish Cause of Death registry, held by the National Board 0f Health and Welfare. All-cause mortality, and cerebrovascular mortality will be recorded for all patients until June 2021, which allow for a minimum follow up of 2 years.

The data management will start when the final data file is received from the National board of Health and Welfare, which is expected in June 2021. Patients without a Swedish personal identification number, and patients with non-specified aetiology or a secondary cause of

For peer review only

intracerebral haemorrhage will be excluded. The final sample is expected to consist of approximately 700 patients with primary intracerebral haemorrhage.

Continuous variables will be either mean-centred, or dichotomized when appropriate. For ordinal variables, categories with limited observations will be merged on the base of clinical reasoning. All variables and data sources are listed in Table 1. The categories of SGPALS will be collapsed into inactive (SGPALS level 1) and physically active (SGPALS level 2-4) for all analyses. NIHSS scores will be categorised as very mild (NIHSS 0-1), mild (NIHSS 2-7), moderate (NIHSS 8-15), and severe (NIHSS ≥16) based on distributional assumptions.[6] The weighted Charlson Comorbidity Index will be calculated, and applied as a four-level ordinal variable using the ICD-10 diagnoses specified in Table 1. The 1-year mortality risk described by Charlson et al. was 12% for patients with 0 points, 26% for 1–2 points, 52% for 3–4 points and 85% for ≥5 points.[25]

*Sample size calculation*

The sample size was estimated using the formula for ordered categorical data suggested by John Whitehead, stated below.[26] The primary outcome measure for the power calculation is the difference in stroke severity on a three-level scale between inactive and physically active patients. Control probabilities were collected from previous research (Table 2).[6] We expect a fraction of 60% physically inactive patients (SGPALS level 1). The sample size estimate was calculated to detect an odds ratio of 0.5 at an alpha level of 0.01 with a power of 80. Based on the power calculation we need a minimum of 628 patients with intracerebral haemorrhage, which is within the expected number of cases.

$$n = 6(Z_{1-\frac{\alpha}{2}} + Z_{\beta})^2 / \left( log(OR)^2 \left[ 1 - \sum_{i=1}^{k} ((\pi_{iE} + \pi_{iC})/2)^3 \right] \right)$$

**Statistical analyses**

Data will be analysed using R, version 4.0.2 (R Core Team. R: A language and environment for statistical computing. R Foundation for Statistical Computing, Vienna, Austria.), and SPSS Statistics for Windows, version 27.0 (released on 2018; IBM Corp., Armonk, NY). All statistical testing will be interpreted at a two-tailed significance level of 0.01 to adjust for multiple comparisons.

*Missing data*

For peer review only

Missing observations for each variable will be explored, and variables with more than 30% will be excluded from the data set. Individuals with missing values on more than 20% of the remaining variables will be excluded from analyses. The number of missing observations in the total data set is expected to be below 3%. Multiple imputation by chained equations will be applied to handle missing data. We will use the predictive mean matching method, originally proposed by Donald B. Rubin,[27] implemented for multiple imputation.[28] The method generates a separate model for each variable with missing data, and creates multiple imputations for each value. The predictive mean algorithm preserves the original data distribution and allows for imputation of continuous, binary, and categorical data.[29] The imputation will be performed separately for patients with primary intracerebral haemorrhage and ischemic stroke, and ordered from the variable with least missing values (monotone). Only variables with a minimum correlation of 0.1 and at least 50% data will be used for prediction. The number of multiple imputations will be set to $m = 20$.

*Propensity scores and matching*

To achieve equipoise between patients with primary intracerebral haemorrhage and ischemic stroke, propensity score matching will be applied. We will include the entire cohort of patients with primary intracerebral haemorrhage, and match them with consecutive ischemic stroke patients, in a 1:2 ratio. The propensity score will be based on sex and potential confounding variables. The rational for the selection of confounders is given below. Here, we assume that confounders for the effect of physical activity on stroke severity will be analogous for primary intracerebral haemorrhage and ischemic stroke. We will conduct generalized boosted regression modelling to calculate the propensity scores.[30] We intend to estimate the average effect of being physically active, i.e. the average treatment effect for the treated will be applied. The number of trees, shrinkage and interaction depth will be tuned by using 10-fold cross validation. We will use the stopping rule es.mean, which uses the absolute standardized bias as balance metric and the mean of the covariate balance metrics to summarize across variables. The propensity score matching will be conducted using the nearest neighbour matching method, with a logistic distance measure and without replacement.[31]

*Regression models*

We will conduct separate models for haemorrhagic and ischemic stroke cases, respectively. All models will be adjusted for potential confounders. The association between pre-stroke physical activity and admission stroke severity will be evaluated using multivariable ordinal regression models.[32] For the ordinal regression, NIHSS categories will be collapsed into very

For peer review only

mild (NIHSS 0-1), mild (NIHSS 2-7) and moderate to severe (NIHSS > 8). A subgroup analysis will be performed for patients with first-time intracerebral haemorrhage. Prior to the analyses, absence of multicollinearity, and the proportional odds assumption will be checked. Diagnostics will be performed using surrogate residuals.[33]

The association between pre-stroke physical activity and all-cause mortality will be evaluated using multivariable Cox Proportional-Hazards models.[34] Additional models will be fitted adjusted for admission stroke severity.[35] Model diagnostics will be applied to evaluate violations of the proportional hazards assumption, random censoring, and functional forms of continuous covariates.[36] Furthermore, outlying and influential observations will be checked for all models.

*Patient and public involvement*

There were no patients or members of the public involved in the planning of this study.

**Confounding variables**

There is limited evidence for the relationship between physical activity and previously known determinants of outcomes following primary intracerebral haemorrhage. However, several factors may confound or mediate the potential impact of physical activity. The covariates included as potential confounders in this study are based on theoretical reasoning, and current evidence. The relationship between pre-stroke physical activity and the main groups of confounders is portrayed in Figure 1.

Degenerative vascular changes are frequent in the aging population. In addition to being a risk factor for all stroke, higher age has been identified as an important predictor of disability, and decreased long-term survival in intracerebral haemorrhage [37,38] A persons age also influence the probability of being physically active,[39] and should therefore be considered a confounding factor. Sex, however, did not significantly influence the severity of haemorrhagic stroke in a meta-analysis of eight population-based stroke incidence studies.[40]

Socioeconomic status-related differences influence haemorrhagic stroke severity, but could partly be explained by differences in comorbidities, disparity in health and care services, ethnicity, stress, job strain and life style factors. Income, education, and living alone have been associated with mortality following the acute phase of stroke.[41] Low income was found to increase the absolute risk of death at 3 months after intracerebral haemorrhage, and the effect was largely mediated by initial stroke severity.[42] In adulthood, socioeconomic variables

For peer review only

are associated with the propensity of being physically active.[43] Among young people, a recent study found that haemorrhagic stroke survivors of African and Hispanic ethnicity have better outcomes compared with those of Caucasian descent.[44] In the current study, country of birth will be used as a surrogate measure for ethnicity.

Active cigarette smoking has been associated with increased stroke severity,[45,46] and premorbid alcohol consumption has been identified as independent determinant of functional status and mortality after intracerebral haemorrhage.[47] Cigarette smoking and physical activity are intuitively incongruent behaviours, and both positive and negative associations has been described.[48] Likewise, alcohol consumption has in some studies been found to be a contributor to sedentary behaviour.[49]

Patients with severe comorbidity may experience worse outcomes following intracerebral haemorrhage, although this has not been thoroughly investigated. One study found that dialysis patients with a medical history of stroke, diabetes and malignancy had a higher risk of mortality following intracerebral haemorrhage.[50] In another study, global comorbidity evaluated by the Charlson comorbidity index was associated with increased risk of 90-day mortality, but not with the length of hospital stay, among immobile patients below 50 years of age.[51] Severe disease may limit a person's ability to be physically active, and thereby confound the effect of pre-stroke physical activity on outcomes following primary intracerebral haemorrhage.

Psychiatric comorbidity has been associated with stroke severity and functional outcomes in a population with both haemorrhagic and ischaemic stroke cases.[52] Physical activity decrease the risk of depression, and a majority of people with depression report sedentary behaviour.[53,54] Similarly, patients with psychotic diseases, such as Schizophrenia, generally have a very low level of physical activity.[55]

Hyperglycaemia and diabetes have been related to worse outcomes after primary intracerebral haemorrhage. Diabetes and high blood glucose in nondiabetic patients have been associated with cerebral complications, and are independent predictors of 30-day mortality, and 3-month mortality following intracerebral haemorrhage.[56] Hyperglycaemia has also been found to decrease short term survival after intracerebral haemorrhage.[57,58] The presence of hyperglycaemia in severe stroke may, however, be a result of an extensive cerebral lesion, causing a phycological increment of cortisol and catecholamines. Regular physical activity promotes blood glucose control in diabetic patients.[59] Still, a large survey of

For peer review only

the U.S. population found that diabetic adults generally engaged in less physical activities compared to non-diabetic adults.

Atrial fibrillation is present in about one third of patients 3 months after intracerebral haemorrhage,[60] and premorbid atrial fibrillation has been found to predict a higher risk of death 3 months after ictus.[61] However, the risk increment seemed to be mediated by the higher prevalence of antithrombotic treatment in the group with atrial fibrillation. There is a complicated relationship between physical activity and the risk of atrial fibrillation. While a moderate amount of physical activity is protective for atrial fibrillation, vigorous physical activity increase the risk of atrial fibrillation in men.[62]

Coagulopathy caused by anticoagulants increase the risk of hematoma expansion, mortality, and disability after intracerebral haemorrhage. Warfarin usage has been associated with increased risk of in-hospital hematoma expansion,[63] and a doubled risk of mortality at 3 months after intracerebral haemorrhage.[64] In an international multicenter pooled analysis, haemorrhagic volume on admission, hematoma expansion, functional status at discharge and 90-day mortality were similar in patients treated with warfarin and non–vitamin K antagonist oral anticoagulation (NOAK), meaning that the risk increment is substantial for treatment with NOAK as well.[65] The propensity of being physically active may be connected to anticoagulant treatment through differences in the prevalence of atrial fibrillation and previous strokes.

The use of lipid lowering drugs has been controversial in patients with intracerebral haemorrhage as hypocholesterolaemia has been identified as a risk factor for intracerebral haemorrhage and hematoma expansion.[66,67] The use of statins has, however, only been correlated to an increased risk of intracerebral haemorrhage in patients with a prior haemorrhagic or lacunar ischemic stroke. On the other hand, several observational studies have reported better outcomes after intracerebral haemorrhage among patients medicated with statins. Premorbid statin usage was associated with a better long-term outcome at 12 months after intracerebral haemorrhage.[68] Another study found that statins reduced mortality and disability without any negative effect on hematoma growth, but the association disappeared when adjusted for potential indication bias.[69] Regular physical activity positively alter the levels of blood lipids, and thereby decrease the probability of receiving statin treatment.[70]

For peer review only

A previous stroke or transient ischaemic attack (TIA) can potentially precondition the brain to endure ischemia, and having gone through a TIA has been associated with lower ischemic stroke severity.[3] Although ischemic tolerance may play an important role in haemorrhagic stroke severity, the influence of a preceding stroke or TIA has not yet been evaluated. Patients diagnosed with stroke or TIA are, however, likely to receive recommendations regarding physical activity in accordance post-stroke guidelines.

In conclusion, the following potential confounders will be collected, and adjusted for: age, ethnicity, income, educational level, comorbidity, medical treatments, alcohol related disorders, drug abuse, and smoking. The comorbidities accounted for are prior TIA, prior stroke, diabetes, psychiatric disorders and Charlson comorbidity index. The medical treatments accounted for are anticoagulant treatment, antihypertensive treatment, antiplatelet treatment, and statin treatment.

## Ethics

Data collection for the PAPSIGOT project was approved by the Regional Ethical Board of Gothenburg on May 4, 2016 (346–16), and by the Swedish Ethical Review Authority (amendment 2021-01109). Research within the framework of quality registers does not require written informed consent from included patients. The Personal Data Act (Swedish law #1998:204, issued 29 April 1998) allows data from medical records to be collected for quality control without receiving a written informed consent.

For peer review only

For peer review only

**Figure legend**

**Figure 1.** Directed acyclic graph for confounders to the effect of physical activity on outcomes after primary intracerebral haemorrhage (stroke severity and post-stroke mortality). Solid lines indicate the relationships between covariates, and the dotted line indicate the causal effect of physical activity.

For peer review only

**Table 1.** Registers and variables included in the study.

| | |
|---|---|
| **The Väststroke register** | |
| Age | Mean-centred. |
| Sex | Female/Male. |
| Physical activity assessment (SGPALS) | Mostly sedentary (Level 1); Light physical activity such as walking ≥ 4 hours/week (Level 2); Moderate physical activity such as running ≥ 2 hours/week (Level 3); Hard physical training for competition sports several times per week (Level 4). |
| Stroke severity assessment (NIHSS) | Very mild (NIHSS 0-1), mild (NIHSS 2-7), moderate (NIHSS 8-15), and severe (NIHSS ≥16) stroke. |
| Acute systolic blood pressure | Dichotomized based on median value. |
| Acute diastolic blood pressure | Dichotomized based on median value. |
| **The Riksstroke register** | |
| Prior Stroke | Yes/No. |
| Prior transient ischaemic attack | Yes/No. |
| Atrial fibrillation | Yes/No. |
| Diabetes | Yes/No. |
| Regular smoking the year before stroke | Yes/No. |
| Antihypertensive treatment | Yes/No. |
| Statin treatment | Yes/No. |
| Antiplatelet treatment | Yes/No. |
| Anticoagulant treatment | Yes/No. |
| **The LISA register** | |
| Country of birth | Born in Sweden/Born outside of Sweden. |
| Income | Ordered in quantiles (Q1 to Q5). |
| Educational level | Primary school (<10 years), secondary school (10-12 years), and postsecondary or university education (>12 years). |
| **The NPR** | |

For peer review only

| | |
|---|---|
| Charlson comorbidity index: Myocardial infarction, Heart failure, Peripheral vascular disease, Dementia, Pulmonary disease, Connective tissue disease, Peptic ulcer disease Liver disease, Diabetes, Hemiplegia or paraplegia, Renal disease, Cancer, HIV/AIDS | Low (0 points), moderate (1-2 points), high (3-4 points), and very high (≥5 points) risk. ICD-10 codes: I21, I22, I25.2, I42, I11.0, I13.0, I13.2 I50, I25.5, I65, I70-I74, I77, I73.9, I79.0, R02, F00-F03, F05.1, J40-J47, J60-J67, M05.0-M05.3, M05.8-M06.0, M06.3, M06.9, M32, M33.2, M34, M35.3, K25-K28, K70.2, K70.3, K71.7, K73, k74.0, K74.2-K74.6, K72.9, K76.6, K76.7, K72.1, E10.1, E10.5, E10.9, E11.1, E11.5, E11.9, E13.1, E13.5, E13.9, E14.1, E14.5, E14.9, E10.2-E10.4, E11.2-E11.4, E13.2-E13.4, E14.2-E14.4, G80.0, G80.2, G81, G82, N01-N08, N11, N14-N19, N25, C00-C76, C90, C97, C91-C95, C81-C86, C88, C77-C80, B20-B24 |
| Depression | Yes/No. ICD-10 codes: F32-F33 |
| Psychotic diseases | Yes/No. ICD-10 codes: F20-F29 |
| Alcohol related disorders | Yes/No. ICD-10 codes: F10, K70.1-K70.4, K70.9, K74.0 |
| Drug abuse | Yes/No. ICD-10 codes: F11, T40.0-T40.4, F12, T40.7, F14, T40.5, F15, T43.6, F16, T40.6, F19 |
| **The Swedish cause of death register** | **ICD-10 code** |
| All-cause mortality | - |
| Cerebrovascular mortality | ICD-10 codes: I60-I69 |

**Table 2.** Frequencies of stroke severity using the National Institutes of Health Stroke Scale (NIHSS), in physically inactive and active patients with stroke (Reinholdsson et al. 2018).[6]

| | No physical activity $\pi_C$ | Physical activity $\pi_E$ | Cumulative Odds ratio |
|---|---|---|---|
| Mild stroke (NIHSS 0-5) | 73.6% | 86.5% | 0.44 |
| Moderate stroke (NIHSS 6-14) | 20.4% | 10.8% | 0.43 |

For peer review only

| | | | |
|---|---|---|---|
| Severe stroke (NIHSS >14) | 6.0% | 2.7% | * |

For peer review only

**Contributorship statement**

A.V has taken part in the planning and design of this study. A.V will collect the data, take part in data analysis, and write the manuscript. D.B has taken part in the design of this study. D.B will take part in the data analysis and revise the manuscript. K.S.S has led the planning and design of this study. K.S.S will assist in the interpretation of data and revise the manuscript.

**Competing interests**

The authors report no conflict of interest

**Funding**

The study was funded by the Swedish Research Council (VR2017-00946), the Swedish Heart and Lung Foundation, the Swedish Brain Foundation, the Swedish Stroke Association, Promobilia, and the Swedish state under an agreement between the Swedish government and the county councils: the ALF agreement (ALFGBG-71980).

For peer review only

For peer review only

## References

1. Labrecque JA, Swanson SA. Target trial emulation: teaching epidemiology and beyond. *Eur J Epidemiol* 2017; **32**(6): 473-5.
2. Thomas L, Peterson ED. The value of statistical analysis plans in observational research: defining high-quality research from the start. *Jama* 2012; **308**(8): 773-4.
3. Deplanque D, Masse I, Lefebvre C, Libersa C, Leys D, Bordet R. Prior TIA, lipid-lowering drug use, and physical activity decrease ischemic stroke severity. *Neurology* 2006; **67**(8): 1403-10.
4. Deplanque D, Masse I, Libersa C, Leys D, Bordet R. Previous leisure-time physical activity dose dependently decreases ischemic stroke severity. *Stroke Research and Treatment* 2012; **2012**: 614925.
5. Krarup LH, Truelsen T, Gluud C, et al. Prestroke physical activity is associated with severity and long-term outcome from first-ever stroke. *Neurology* 2008; **71**(17): 1313-8.
6. Reinholdsson M, Palstam A, Sunnerhagen KS. Prestroke physical activity could influence acute stroke severity (part of PAPSIGOT). *Neurology* 2018; **91**(16): e1461-e7.
7. Ricciardi AC, Lopez-Cancio E, Perez de la Ossa N, et al. Prestroke physical activity is associated with good functional outcome and arterial recanalization after stroke due to a large vessel occlusion. *Cerebrovascular Diseases* 2014; **37**(4): 304-11.

8. Wen CP, Liu CH, Jeng JS, et al. Pre-stroke physical activity is associated with fewer post-stroke complications, lower mortality and a better long-term outcome. *European Journal of Neurology* 2017; **24**(12): 1525-31.
9. Mediano MFF, Mok Y, Coresh J, et al. Prestroke Physical Activity and Adverse Health Outcomes After Stroke in the Atherosclerosis Risk in Communities Study. *Stroke* 2021: Strokeaha120032695.
10. Bell CL, LaCroix A, Masaki K, et al. Prestroke factors associated with poststroke mortality and recovery in older women in the Women's Health Initiative. *Journal of the American Geriatrics Society* 2013; **61**(8): 1324-30.
11. Morovatdar N, Di Napoli M, Stranges S, et al. Regular physical activity postpones age of occurrence of first-ever stroke and improves long-term outcomes. *Neurol Sci* 2020.
12. Li HX, Wang PS, Tian GL, Xu WL, Liu D, Wu J. A seven-year follow-up analysis on prognosis factors of stroke patients. *Chung Hua Liu Hsing Ping Hsueh Tsa Chih* 2005; **26**(9): 716-9.
13. Stroud N, Mazwi TM, Case LD, et al. Prestroke physical activity and early functional status after stroke. *J Neurol Neurosurg Psychiatry* 2009; **80**(9): 1019-22.
14. Ursin MH, Ihle-Hansen H, Fure B, Tveit A, Bergland A. Effects of premorbid physical activity on stroke severity and post-stroke functioning. *Journal of Rehabilitation Medicine* 2015; **47**(7): 612-7.
15. Decourcelle A, Moulin S, Sibon I, et al. Influence of previous physical activity on the outcome of patients treated by thrombolytic therapy for stroke. *Journal of Neurology* 2015; **262**(11): 2513-9.
16. Redfors P, Isaksen D, Lappas G, et al. Living alone predicts mortality in patients with ischemic stroke before 70 years of age: a long-term prospective follow-up study. *BMC Neurology* 2016; **16**: 80.
17. Christensen MC, Mayer S, Ferran J-M. Quality of Life After Intracerebral Hemorrhage. *Stroke* 2009; **40**(5): 1677-82.
18. Qureshi AI, Mendelow AD, Hanley DF. Intracerebral haemorrhage. *Lancet (London, England)* 2009; **373**(9675): 1632-44.
19. Cordonnier C, Demchuk A, Ziai W, Anderson CS. Intracerebral haemorrhage: current approaches to acute management. *Lancet (London, England)* 2018; **392**(10154): 1257-68.
20. Krishnamurthi RV, Ikeda T, Feigin VL. Global, Regional and Country-Specific Burden of Ischaemic Stroke, Intracerebral Haemorrhage and Subarachnoid Haemorrhage: A Systematic Analysis of the Global Burden of Disease Study 2017. *Neuroepidemiology* 2020; **54**(2): 171-9.
21. Gamble C, Krishan A, Stocken D, et al. Guidelines for the Content of Statistical Analysis Plans in Clinical Trials. *Jama* 2017; **318**(23): 2337-43.
22. Saltin B, Grimby G. Physiological analysis of middle-aged and old former athletes. Comparison with still active athletes of the same ages. *Circulation* 1968; **38**(6): 1104-15.
23. Grimby G, Börjesson M, Jonsdottir IH, Schnohr P, Thelle DS, Saltin B. The "Saltin-Grimby Physical Activity Level Scale" and its application to health research. *Scand J Med Sci Sports* 2015; **25 Suppl 4**: 119-25.
24. Muir KW, Weir CJ, Murray GD, Povey C, Lees KR. Comparison of neurological scales and scoring systems for acute stroke prognosis. *Stroke* 1996; **27**(10): 1817-20.
25. Charlson ME, Pompei P, Ales KL, MacKenzie CR. A new method of classifying prognostic comorbidity in longitudinal studies: development and validation. *J Chronic Dis* 1987; **40**(5): 373-83.
26. Whitehead J. Sample size calculations for ordered categorical data. *Stat Med* 1993; **12**(24): 2257-71.
27. Rubin DB. Statistical Matching Using File Concatenation With Adjusted Weights and Multiple Imputations. *Journal of Business & Economic Statistics* 1986; **4**(1): 87-94.
28. van Buuren S, Groothuis-Oudshoorn K. mice: Multivariate Imputation by Chained Equations in R. *2011* 2011; **45**(3): 67.

29. Vink G, Frank LE, Pannekoek J, Buuren S. Predictive mean matching imputation of semicontinuous variables. *Statistica Neerlandica* 2014; **68**(1): 61-90.
30. McCaffrey DF, Ridgeway G, Morral AR. Propensity score estimation with boosted regression for evaluating causal effects in observational studies. *Psychol Methods* 2004; **9**(4): 403-25.
31. Ho DE, Imai K, King G, Stuart EA. Matching as Nonparametric Preprocessing for Reducing Model Dependence in Parametric Causal Inference. *Political Analysis* 2007; **15**(3): 199-236.
32. Christensen R. Cumulative Link Models for Ordinal Regression with the R Package ordinal. 2018; 2018.
33. Liu D, Zhang H. Residuals and Diagnostics for Ordinal Regression Models: A Surrogate Approach. *Journal of the American Statistical Association* 2018; **113**: 1-10.
34. Cox DR. Regression Models and Life-Tables. *Journal of the Royal Statistical Society: Series B (Methodological)* 1972; **34**(2): 187-202.
35. Borgan Ø. Modeling Survival Data: Extending the Cox Model. Terry M. Therneau and Patricia M. Grambsch, Springer-Verlag, New York, 2000. ISBN 0-387-98784-3. *Statistics in Medicine* 2001; **20**(13): 2053-4.
36. Xue Y, Schifano ED. Diagnostics for the Cox model. *CSAM* 2017; **24**(6): 583-604.
37. Bagg S, Pombo AP, Hopman W. Effect of age on functional outcomes after stroke rehabilitation. *Stroke* 2002; **33**(1): 179-85.
38. Sacco S, Marini C, Toni D, Olivieri L, Carolei A. Incidence and 10-year survival of intracerebral hemorrhage in a population-based registry. *Stroke* 2009; **40**(2): 394-9.
39. King AC, King DK. Physical Activity for an Aging Population. *Public Health Reviews* 2010; **32**(2): 401-26.
40. Phan HT, Reeves MJ, Blizzard CL, et al. Sex Differences in Severity of Stroke in the INSTRUCT Study: a Meta-Analysis of Individual Participant Data. *J Am Heart Assoc* 2019; **8**(1): e010235.
41. Lindmark A, Glader E-L, Asplund K, Norrving B, Eriksson M. Socioeconomic Disparities in Stroke Case Fatality – Observations from Riks-Stroke, the Swedish Stroke Register. *International Journal of Stroke* 2014; **9**(4): 429-36.
42. Lindmark A, Norrving B, Eriksson M. Socioeconomic status and survival after stroke – using mediation and sensitivity analyses to assess the effect of stroke severity and unmeasured confounding. *BMC Public Health* 2020; **20**(1): 554.
43. O'Donoghue G, Kennedy A, Puggina A, et al. Socio-economic determinants of physical activity across the life course: A "DEterminants of DIet and Physical ACtivity" (DEDIPAC) umbrella literature review. *PLoS One* 2018; **13**(1): e0190737.
44. Miyares LC, Falcone GJ, Leasure A, et al. Race/ethnicity influences outcomes in young adults with supratentorial intracerebral hemorrhage. *Neurology* 2020; **94**(12): e1271-e80.
45. Edjoc RK, Reid RD, Sharma M, Fang J. The prognostic effect of cigarette smoking on stroke severity, disability, length of stay in hospital, and mortality in a cohort with cerebrovascular disease. *Journal of stroke and cerebrovascular diseases : the official journal of National Stroke Association* 2013; **22**(8): e446-54.
46. Weng WC, Huang WY, Chien YY, et al. The impact of smoking on the severity of acute ischemic stroke. *J Neurol Sci* 2011; **308**(1-2): 94-7.
47. Juvela S. Risk factors for impaired outcome after spontaneous intracerebral hemorrhage. *Arch Neurol* 1995; **52**(12): 1193-200.
48. Kaczynski AT, Manske SR, Mannell RC, Grewal K. Smoking and physical activity: a systematic review. *Am J Health Behav* 2008; **32**(1): 93-110.
49. Vancampfort D, De Hert M, Stubbs B, et al. A systematic review of physical activity correlates in alcohol use disorders. *Arch Psychiatr Nurs* 2015; **29**(4): 196-201.
50. Lin CY, Chien CC, Chen HA, et al. The impact of comorbidity on survival after hemorrhagic stroke among dialysis patients: a nationwide population-based study. *BMC Nephrol* 2014; **15**: 186.

For peer review only

51. Liu H, Wu X, Cao J, et al. Effect of Comorbidity Assessed by the Charlson Comorbidity Index on the Length of Stay and Mortality Among Immobile Hemorrhagic Stroke Patients Younger Than 50 Years. *Frontiers in neurology* 2020; **11**: 487.
52. Hoyer C, Schmidt HL, Kranaster L, Alonso A. Impact of psychiatric comorbidity on the severity, short-term functional outcome, and psychiatric complications after acute stroke. *Neuropsychiatr Dis Treat* 2019; **15**: 1823-31.
53. Camacho TC, Roberts RE, Lazarus NB, Kaplan GA, Cohen RD. Physical Activity and Depression: Evidence from the Alameda County Study. *American Journal of Epidemiology* 1991; **134**(2): 220-31.
54. Roshanaei-Moghaddam B, Katon WJ, Russo J. The longitudinal effects of depression on physical activity. *General Hospital Psychiatry* 2009; **31**(4): 306-15.
55. Stubbs B, Firth J, Berry A, et al. How much physical activity do people with schizophrenia engage in? A systematic review, comparative meta-analysis and meta-regression. *Schizophrenia Research* 2016; **176**(2): 431-40.
56. Passero S, Ciacci G, Ulivelli M. The influence of diabetes and hyperglycemia on clinical course after intracerebral hemorrhage. *Neurology* 2003; **61**(10): 1351-6.
57. Fogelholm R, Murros K, Rissanen A, Avikainen S. Admission blood glucose and short term survival in primary intracerebral haemorrhage: a population based study. *J Neurol Neurosurg Psychiatry* 2005; **76**(3): 349-53.
58. Kimura K, Iguchi Y, Inoue T, et al. Hyperglycemia independently increases the risk of early death in acute spontaneous intracerebral hemorrhage. *J Neurol Sci* 2007; **255**(1-2): 90-4.
59. Trovati M, Carta Q, Cavalot F, et al. Influence of physical training on blood glucose control, glucose tolerance, insulin secretion, and insulin action in non-insulin-dependent diabetic patients. *Diabetes Care* 1984; **7**(5): 416-20.
60. Horstmann S, Rizos T, Jenetzky E, Gumbinger C, Hacke W, Veltkamp R. Prevalence of atrial fibrillation in intracerebral hemorrhage. *European Journal of Neurology* 2014; **21**(4): 570-6.
61. Roquer J, Vivanco-Hidalgo RM, Prats-Sánchez LL, et al. Interaction of atrial fibrillation and antithrombotics on outcome in intracerebral hemorrhage. *Neurology* 2019; **93**(19): e1820-e9.
62. Nattel S. Physical activity and atrial fibrillation risk: it's complicated; and sex is critical. *European heart journal* 2020; **41**(15): 1487-9.
63. Flibotte JJ, Hagan N, O'Donnell J, Greenberg SM, Rosand J. Warfarin, hematoma expansion, and outcome of intracerebral hemorrhage. *Neurology* 2004; **63**(6): 1059-64.
64. Rosand J, Eckman MH, Knudsen KA, Singer DE, Greenberg SM. The effect of warfarin and intensity of anticoagulation on outcome of intracerebral hemorrhage. *Archives of internal medicine* 2004; **164**(8): 880-4.
65. Wilson D, Seiffge DJ, Traenka C, et al. Outcome of intracerebral hemorrhage associated with different oral anticoagulants. *Neurology* 2017; **88**(18): 1693-700.
66. Puddey IB. Low serum cholesterol and the risk of cerebral haemorrhage. *Atherosclerosis* 1996; **119**(1): 1-6.
67. Rodriguez-Luna D, Rubiera M, Ribo M, et al. Serum low-density lipoprotein cholesterol level predicts hematoma growth and clinical outcome after acute intracerebral hemorrhage. *Stroke* 2011; **42**(9): 2447-52.
68. Winkler J, Shoup JP, Czap A, et al. Long-term improvement in outcome after intracerebral hemorrhage in patients treated with statins. *Journal of stroke and cerebrovascular diseases : the official journal of National Stroke Association* 2013; **22**(8): e541-5.
69. Siddiqui FM, Langefeld CD, Moomaw CJ, et al. Use of Statins and Outcomes in Intracerebral Hemorrhage Patients. *Stroke* 2017; **48**(8): 2098-104.
70. Durstine JL, Grandjean PW, Cox CA, Thompson PD. Lipids, lipoproteins, and exercise. *J Cardiopulm Rehabil* 2002; **22**(6): 385-98.

For peer review only

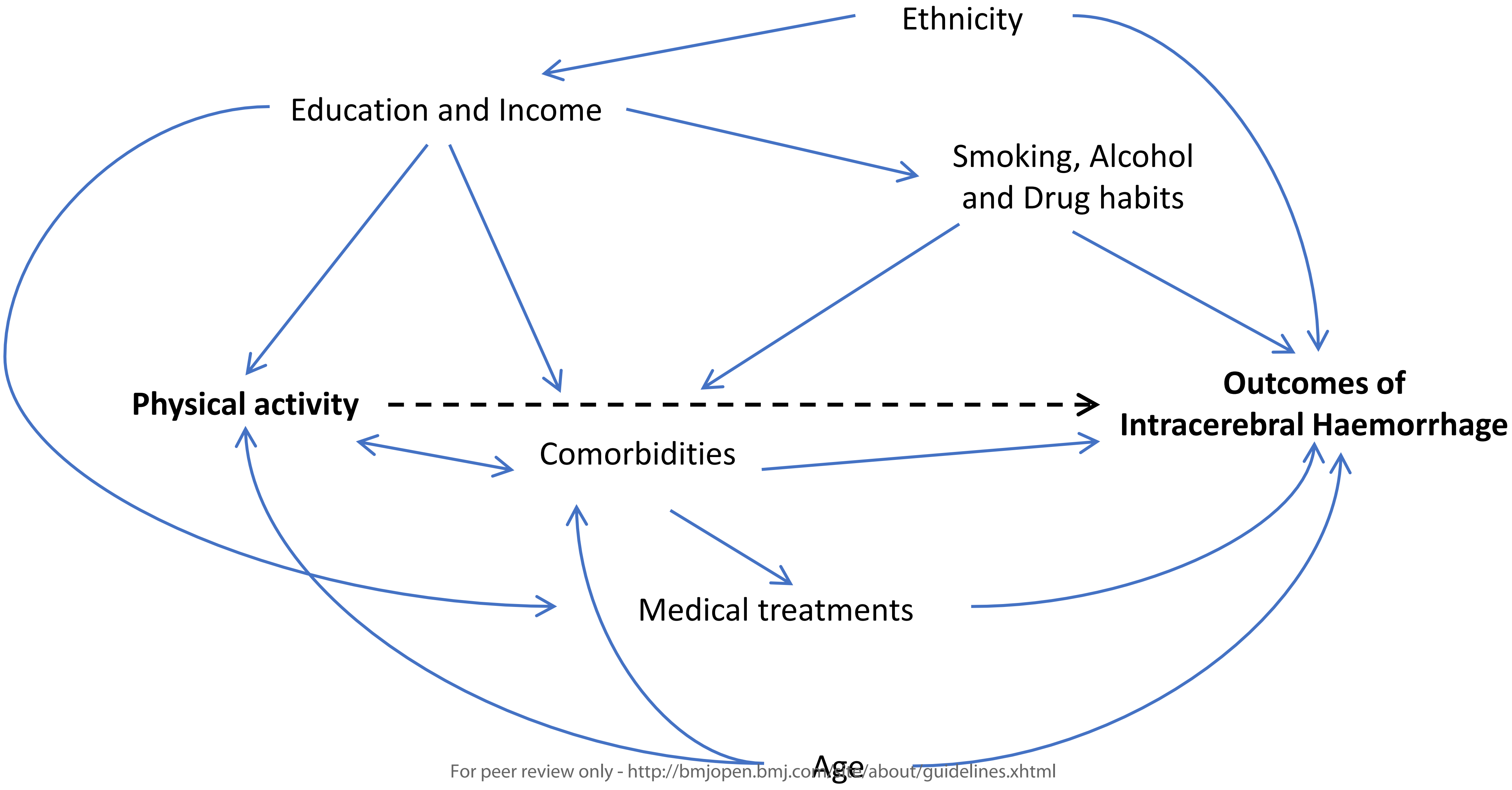
Ethnicity
Education and Income
Smoking, Alcohol and Drug habits
Physical activity
Outcomes of Intracerebral Haemorrhage
Comorbidities
Medical treatments
Age

BMJ Open

# Pre-stroke physical activity and outcomes after intracerebral haemorrhage in comparison to ischemic stroke: protocol for a matched cohort study (part of PAPSIGOT)

| | |
|---|---|
| Journal: | *BMJ Open* |
| Manuscript ID | bmjopen-2021-053067.R1 |
| Article Type: | Protocol |
| Date Submitted by the Author: | 23-Sep-2021 |
| Complete List of Authors: | Viktorisson, Adam; Goteborgs universitet Institutionen for neurovetenskap och fysiologi, Rehabilitation medicine<br>Buvarp, Dongni; University of Gothenburg<br>Sunnerhagen, Katharina; Univ of Gothenburg, Inst of Neuroscience and Physiology |
| <b>Primary Subject Heading</b>: | Neurology |
| Secondary Subject Heading: | Research methods, Public health |
| Keywords: | STATISTICS & RESEARCH METHODS, STROKE MEDICINE, Protocols & guidelines < HEALTH SERVICES ADMINISTRATION & MANAGEMENT, PREVENTIVE MEDICINE |
| | |

SCHOLARONE™
Manuscripts

*I, the Submitting Author has the right to grant and does grant on behalf of all authors of the Work (as defined in the below author licence), an exclusive licence and/or a non-exclusive licence for contributions from authors who are: i) UK Crown employees; ii) where BMJ has agreed a CC-BY licence shall apply, and/or iii) in accordance with the terms applicable for US Federal Government officers or employees acting as part of their official duties; on a worldwide, perpetual, irrevocable, royalty-free basis to BMJ Publishing Group Ltd ("BMJ") its licensees and where the relevant Journal is co-owned by BMJ to the co-owners of the Journal, to publish the Work in this journal and any other BMJ products and to exploit all rights, as set out in our licence.*

*The Submitting Author accepts and understands that any supply made under these terms is made by BMJ to the Submitting Author unless you are acting as an employee on behalf of your employer or a postgraduate student of an affiliated institution which is paying any applicable article publishing charge ("APC") for Open Access articles. Where the Submitting Author wishes to make the Work available on an Open Access basis (and intends to pay the relevant APC), the terms of reuse of such Open Access shall be governed by a Creative Commons licence – details of these licences and which Creative Commons licence will apply to this Work are set out in our licence referred to above.*

*Other than as permitted in any relevant BMJ Author's Self Archiving Policies, I confirm this Work has not been accepted for publication elsewhere, is not being considered for publication elsewhere and does not duplicate material already published. I confirm all authors consent to publication of this Work and authorise the granting of this licence.*

For peer review only

# Pre-stroke physical activity and outcomes after intracerebral haemorrhage in comparison to ischemic stroke: protocol for a matched cohort study (part of PAPSIGOT)

Adam Viktorisson (MD) [1,*], Dongni Buvarp (MD, PhD) [1], Katharina S Sunnerhagen (MD, PhD) [1]

1. Institute of Neuroscience and Physiology, Rehabilitation Medicine, Sahlgrenska Academy, University of Gothenburg, and the Sahlgrenska University Hospital, Gothenburg, Sweden.

**Corresponding author:** Adam Viktorisson
Sahlgrenska Academy, Institute of Neuroscience and Physiology,
University of Gothenburg
Per Dubbsgatan 14, 3rd floor
PO Box 430
SE 405 30, Gothenburg, Sweden.
E-mail address: adam.viktorisson@gu.se
Phone: +46-31-342 2803

**Principal investigator:** Prof. Katharina Stibrant Sunnerhagen
Sahlgrenska Academy, Institute of Neuroscience and Physiology,
University of Gothenburg
E-mail address: ks.sunnerhagen@neuro.gu.se

**SAP version number and date:** version 1.0 2021-05-02

## Abstract

*Introduction*

Piling evidence suggests that a higher level of pre-stroke physical activity can decrease stroke severity, and reduce the risk of post-stroke mortality. However, prior studies have only included ischemic stroke cases, or a majority of such. We aim to investigate how premorbid physical activity affect admission stroke severity and post stroke mortality in patients with intracerebral haemorrhage, compared to ischemic stroke. A prespecified analysis plan counteract some inherent biases in observational studies, and promotes transparency.

*Methods and analysis*

For peer review only

This is a statistical analysis protocol for a matched cohort study, including all adult patients with intracerebral haemorrhage, and matched ischemic stroke controls, treated at Sahlgrenska University Hospital in Sweden between November 1, 2014 and June 30, 2019. All patients have been identified in the Väststroke register, and the data file has been sent for merging with national registries. The follow-up of time for survival will be approximately 2 to 7 years. The sample size calculation indicates that a minimum of 628 patients with intracerebral haemorrhage is needed for power of 80% at an alpha level of 0.01. Multiple imputation by chained equations will be used to handle missing data. The entire cohort of patients with intracerebral haemorrhage will be matched with consecutive ischemic stroke controls (1:3 ratio) using nearest neighbour propensity score matching. The association between pre-stroke physical activity and admission stroke severity will be evaluated using multivariable ordinal regression models, and risk for all-cause mortality will be analysed using multivariable Cox Proportional-Hazards models. Potential confounders include age, ethnicity, income, educational level, comorbidity, medical treatments, alcohol related disorders, drug abuse, and smoking.

*Ethics*

Data collection for the Physical Activity Pre-Stroke In GOThenburg (PAPSIGOT) project was approved by the Regional Ethical Board on May 4, 2016. An additional application was approved by the National Ethical Review Authority on July 7, 2021.

**Strengths and limitations of this study**

- A prespecified sample size calculation and statistical analysis plan decrease bias in observational research.
- The novel relationship between pre-stroke physical activity and outcomes after intracerebral haemorrhage will be studied in a large sample.
- Comprehensive covariate adjustment will enhance the reliability of the results.
- The retrospective analysis of registry data is a limitation of the study.
- Self-reported assessments of pre-stroke physical activity introduce recall bias, which is another limitation of the study.

**Introduction**

For peer review only

Observational studies are of great value in cases when a randomized controlled trial would be unethical, impractical or untimely. Observational studies are, however, subjected to several types of selection and reporting bias, which can lead to a lack in reproducibility. The reliability of observational data can be enhanced by emulating principal design aspects of randomized trials.[1] Pre-specified eligibility criteria, a priori sample size estimation, definition of exposure and outcome measures, a standardised follow-up period, and a statistical analysis plan may counterbalance some inherent limitations of the observational study design, and promote transparency.[2]

Physical activity is an example of exposure that is hard to study in a randomized, controlled setting. The relationship between premorbid physical activity and admission stroke severity, as well as post-stroke mortality has been analysed in several observational studies, with varying results. The majority of prior studies have found that pre-stroke physical activity reduce stroke severity,[3-8] in-hospital mortality,[8] cardiovascular mortality,[9] and all-cause mortality.[9-12] However, some studies found no associations between physical activity and stroke severity,[13,14] or post-stroke mortality after covariate adjustment.[15,16] These studies differ in data collection strategies, sample sizes, physical activity assessments, and statistical analyses. Moreover, none of the prior studies have investigated the association between pre-stroke physical activity and stroke severity or mortality in the subgroup of patients with intracerebral haemorrhage.

Here, we present a statistical analysis plan, and give the rationale for an observational, matched cohort study part of the Physical Activity Pre-Stroke In GOThenburg (PAPSIGOT) project. We also provide a narrative discussion for potential confounding factors to the effect of pre-stroke physical activity on outcomes after intracerebral haemorrhage. This study protocol follows the guidelines for reporting of statistical analysis plans in clinical trials, published in the Journal of the American Medical Association.[17]

### Research objectives

(1) To investigate the relationship between pre-stroke physical activity and admission stroke severity in patients with intracerebral haemorrhage, compared to patients with ischemic stroke. (2) To investigate how pre-stroke physical activity influence the risk of mortality after intracerebral haemorrhage, in comparison to ischemic stroke.

### Methods

For peer review only

This is a statistical analysis protocol for a matched cohort study. Pre-stroke physical activity is the main independent variable. Admission stroke severity, and all-cause mortality are the outcome variables of interest. All adult patients with intracerebral haemorrhage, and ischaemic stroke, treated at Sahlgrenska University Hospital between November 1, 2014 and June 30, 2019 have been identified in the Väststroke register. The comprehensive stroke units at Sahlgrenska University Hospital has a catchment area of 700,000 people, and stroke patients in nearby regions requiring thrombectomy or neurosurgical monitoring are transported to this hospital. All persons with stroke in Sweden have equal access to tax-funded healthcare services, which ensures the representativeness of this sample.

*Data collection and management*

Pre-stroke physical activity and acute stroke severity assessments were collected from the Väststroke register, and cross-references with medical records to minimize missing data. Physical activity was assessed using the four-level Saltin-Grimby Physical Activity Level Scale (SGPALS).[18,19] Acute stroke severity was assessed using the National Institutes of Health Stroke Scale (NIHSS).[20] The data collected from the Väststroke register has been sent for merging with national registries. Information regarding pre-stroke conditions and treatments will be collected from the Swedish National Stroke Registry (Riksstroke). Country of birth will be collected from the Swedish Multi-Generation register, and socioeconomic variables will be collected from the Longitudinal Integration Database for Health Insurance and Labour Market Studies (LISA) held by Statistics Sweden. Comorbidities will be collected from the National Patient Registry (NPR), and mortality rates will be collected from the Swedish Cause of Death registry, held by the National Board of Health and Welfare. All-cause mortality, and cerebrovascular mortality will be recorded for all patients until June 2021, which allow for a minimum follow up of 2 years.

The data management started on May 17, 2021 after submission of this protocol. Patients without a Swedish personal identification number, and patients with non-specified aetiology will be excluded. The final sample is will consist of 763 patients with intracerebral haemorrhage.

Continuous variables will be either mean-centred, or dichotomized when appropriate. For ordinal variables, categories with limited observations will be merged on the base of clinical reasoning. All variables and data sources are listed in Table 1. The categories of SGPALS will be collapsed into inactive (SGPALS level 1) and physically active (SGPALS level 2-4) for all analyses. NIHSS scores will be categorised based on distributional assumptions.[6] The

For peer review only

weighted Charlson Comorbidity Index will be calculated, and applied as a four-level ordinal variable using the ICD-10 diagnoses specified in Table 1. The 1-year mortality risk described by Charlson et al. was 12% for patients with 0 points, 26% for 1–2 points, 52% for 3–4 points and 85% for ≥5 points.[21]

*Sample size calculation*

The sample size was estimated using the formula for ordered categorical data suggested by John Whitehead, stated below.[22] The primary outcome measure for the power calculation is the difference in stroke severity on a three-level scale between inactive and physically active patients. Control probabilities were collected from previous research (Table 2).[6] We expect a fraction of 60% physically inactive patients (SGPALS level 1). The sample size estimate was calculated to detect an odds ratio of 2.0 at an alpha level of 0.01 with a power of 80.[6] Based on the power calculation we need a minimum of 628 patients with intracerebral haemorrhage, which is within the expected number of cases.

$$n = 6(Z_{1-\frac{\alpha}{2}} + Z_{\beta})^2 / \left( log(OR)^2 \left[ 1 - \sum_{i=1}^{k} ((\pi_{iE} + \pi_{iC})/2)^3 \right] \right)$$

**Statistical analyses**

Data will be analysed using R, version 4.0.2 (R Core Team. R: A language and environment for statistical computing. R Foundation for Statistical Computing, Vienna, Austria.), and SPSS Statistics for Windows, version 27.0 (released on 2018; IBM Corp., Armonk, NY). All statistical testing will be interpreted at a two-tailed significance level of 0.01 to adjust for multiple comparisons.

*Missing data*

Missing observations for each variable will be explored, and variables with more than 30% will be excluded from the data set. Individuals with missing values on more than 20% of the remaining variables will be excluded from analyses. The number of missing observations in the total data set is expected to be below 3%. Multiple imputation by chained equations will be applied to handle missing data. We will use the predictive mean matching method, originally proposed by Donald B. Rubin,[23] implemented for multiple imputation.[24] The method generates a separate model for each variable with missing data, and creates multiple imputations for each value. The predictive mean algorithm preserves the original data distribution and allows for imputation of continuous, binary, and categorical data.[25] The imputation will be performed separately for patients with intracerebral haemorrhage and

For peer review only

ischemic stroke, and ordered from the variable with least missing values (monotone). Only variables with a minimum correlation of 0.1 and at least 50% data will be used for prediction. The number of multiple imputations will be set to $m = 20$.

*Propensity scores and matching*

To achieve equipoise between patients with intracerebral haemorrhage and ischemic stroke, propensity score matching will be applied. We will include the entire cohort of patients with intracerebral haemorrhage, and match them with consecutive ischemic stroke patients, in a 1:3 ratio. The propensity score will be based on sex and potential confounding variables. The rational for the selection of confounders is given below. Here, we assume that confounders for the effect of physical activity on stroke severity will be analogous for intracerebral haemorrhage and ischemic stroke. We will conduct generalized boosted regression modelling to calculate the propensity scores.[26] We intend to estimate the average effect of being physically active, i.e. the average treatment effect for the treated will be applied. The number of trees, shrinkage and interaction depth will be tuned by using 10-fold cross validation. We will use the stopping rule es.mean, which uses the absolute standardized bias as balance metric and the mean of the covariate balance metrics to summarize across variables. The propensity score matching will be conducted using the nearest neighbour matching method, with a logistic distance measure and without replacement.[27]

*Regression models*

We will conduct separate models for haemorrhagic and ischemic stroke cases, respectively. All models will be adjusted for potential confounders. The association between pre-stroke physical activity and admission stroke severity will be evaluated using multivariable ordinal regression models.[28] For the ordinal regression, NIHSS scores will be trichotomized. A subgroup analysis will be performed for patients with first-time intracerebral haemorrhage. Prior to the analyses, absence of multicollinearity, and the proportional odds assumption will be checked. Diagnostics will be performed using surrogate residuals.[29]

The association between pre-stroke physical activity and all-cause mortality will be evaluated using multivariable Cox Proportional-Hazards models.[30] Additional models will be fitted adjusted for admission stroke severity.[31] Model diagnostics will be applied to evaluate violations of the proportional hazards assumption, random censoring, and functional forms of continuous covariates.[32] Furthermore, outlying and influential observations will be checked for all models.

For peer review only

*Patient and public involvement*

There were no patients or members of the public involved in the planning of this study.

**Confounding variables**

There is limited evidence for the relationship between physical activity and previously known determinants of outcomes following intracerebral haemorrhage. However, several factors may confound or mediate the potential impact of physical activity. The covariates included as potential confounders in this study are based on theoretical reasoning, and current evidence. The relationship between pre-stroke physical activity and the main groups of confounders is portrayed in Figure 1.

Degenerative vascular changes are frequent in the aging population. In addition to being a risk factor for all stroke, higher age has been identified as an important predictor of disability, and decreased long-term survival in intracerebral haemorrhage [33,34] A persons age also influence the probability of being physically active,[35] and should therefore be considered a confounding factor. Sex, however, did not significantly influence the severity of haemorrhagic stroke in a meta-analysis of eight population-based stroke incidence studies.[36]

Socioeconomic status-related differences influence haemorrhagic stroke severity, but could partly be explained by differences in comorbidities, disparity in health and care services, ethnicity, stress, job strain and life style factors. Income, education, and living alone have been associated with mortality following the acute phase of stroke.[37] Low income was found to increase the absolute risk of death at 3 months after intracerebral haemorrhage, and the effect was largely mediated by initial stroke severity.[38] In adulthood, socioeconomic variables are associated with the propensity of being physically active.[39] Among young people, a recent study found that haemorrhagic stroke survivors of African and Hispanic ethnicity have better outcomes compared with those of Caucasian descent.[40] In the current study, country of birth will be used as a surrogate measure for ethnicity.

Active cigarette smoking has been associated with increased stroke severity,[41,42] and premorbid alcohol consumption has been identified as independent determinant of functional status and mortality after intracerebral haemorrhage.[43] Cigarette smoking and physical activity are intuitively incongruent behaviours, and both positive and negative associations has been described.[44] Likewise, alcohol consumption has in some studies been found to be a contributor to sedentary behaviour.[45]

For peer review only

Patients with severe comorbidity may experience worse outcomes following intracerebral haemorrhage, although this has not been thoroughly investigated. One study found that dialysis patients with a medical history of stroke, diabetes and malignancy had a higher risk of mortality following intracerebral haemorrhage.[46] In another study, global comorbidity evaluated by the Charlson comorbidity index was associated with increased risk of 90-day mortality, but not with the length of hospital stay, among immobile patients below 50 years of age.[47] Severe disease may limit a person's ability to be physically active, and thereby confound the effect of pre-stroke physical activity on outcomes following intracerebral haemorrhage.

Psychiatric comorbidity has been associated with stroke severity and functional outcomes in a population with both haemorrhagic and ischaemic stroke cases.[48] Physical activity decrease the risk of depression, and a majority of people with depression report sedentary behaviour.[49,50] Similarly, patients with psychotic diseases, such as Schizophrenia, generally have a very low level of physical activity.[51]

Hyperglycaemia and diabetes have been related to worse outcomes after intracerebral haemorrhage. Diabetes and high blood glucose in nondiabetic patients have been associated with cerebral complications, and are independent predictors of 30-day mortality, and 3-month mortality following intracerebral haemorrhage.[52] Hyperglycaemia has also been found to decrease short term survival after intracerebral haemorrhage.[53,54] The presence of hyperglycaemia in severe stroke may, however, be a result of an extensive cerebral lesion, causing a phycological increment of cortisol and catecholamines. Regular physical activity promotes blood glucose control in diabetic patients.[55] Still, a large survey of the U.S. population found that diabetic adults generally engaged in less physical activities compared to non-diabetic adults.

Atrial fibrillation is present in about one third of patients 3 months after intracerebral haemorrhage,[56] and premorbid atrial fibrillation has been found to predict a higher risk of death 3 months after ictus.[57] However, the risk increment seemed to be mediated by the higher prevalence of antithrombotic treatment in the group with atrial fibrillation. There is a complicated relationship between physical activity and the risk of atrial fibrillation. While a moderate amount of physical activity is protective for atrial fibrillation, vigorous physical activity increase the risk of atrial fibrillation in men.[58]

For peer review only

Coagulopathy caused by anticoagulants increase the risk of hematoma expansion, mortality, and disability after intracerebral haemorrhage. Warfarin usage has been associated with increased risk of in-hospital hematoma expansion,[59] and a doubled risk of mortality at 3 months after intracerebral haemorrhage.[60] In an international multicenter pooled analysis, haemorrhagic volume on admission, hematoma expansion, functional status at discharge and 90-day mortality were similar in patients treated with warfarin and non–vitamin K antagonist oral anticoagulation (NOAK), meaning that the risk increment is substantial for treatment with NOAK as well.[61] The propensity of being physically active may be connected to anticoagulant treatment through differences in the prevalence of atrial fibrillation and previous strokes.

The use of lipid lowering drugs has been controversial in patients with intracerebral haemorrhage as hypocholesterolaemia has been identified as a risk factor for intracerebral haemorrhage and hematoma expansion.[62,63] Low levels of serum lipids were also independently associated with a poor 3 months prognosis after ICH in women.[64] The use of statins has, however, only been correlated to an increased risk of intracerebral haemorrhage in patients with a prior haemorrhagic or lacunar ischemic stroke. On the other hand, several observational studies have reported better outcomes after intracerebral haemorrhage among patients medicated with statins. Premorbid statin usage was associated with a better long-term outcome at 12 months after intracerebral haemorrhage.[65] Another study found that statins reduced mortality and disability without any negative effect on hematoma growth, but the association disappeared when adjusted for potential indication bias.[66] Regular physical activity positively alter the levels of blood lipids, and may thereby decrease the probability of receiving statin treatment.[67]

A previous stroke or transient ischaemic attack (TIA) can potentially precondition the brain to endure ischemia, and having gone through a TIA has been associated with lower ischemic stroke severity.[3] Although ischemic tolerance may play an important role in haemorrhagic stroke severity, the influence of a preceding stroke or TIA has not yet been evaluated. Patients diagnosed with stroke or TIA are, however, likely to receive recommendations regarding physical activity in accordance post-stroke guidelines.

In conclusion, the following potential confounders will be collected, and adjusted for: age, ethnicity, income, educational level, comorbidity, medical treatments, alcohol related disorders, drug abuse, and smoking. The comorbidities accounted for are prior TIA, prior stroke, diabetes, psychiatric disorders and Charlson comorbidity index. The medical

For peer review only

treatments accounted for are anticoagulant treatment, antihypertensive treatment, antiplatelet treatment, and statin treatment.

### Ethics

Data collection for the PAPSIGOT project was approved by the Regional Ethical Board of Gothenburg on May 4, 2016 (346–16). An additional application was approved by the Swedish Ethical Review Authority on July 7, 2021 (2021-03324). Research within the framework of quality registers does not require written informed consent from included patients. The Personal Data Act (Swedish law #1998:204, issued 29 April 1998) allows data from medical records to be collected for quality control without receiving a written informed consent.

For peer review only

For peer review only

**Figure legend**

**Figure 1.** Directed acyclic graph for confounders to the effect of physical activity on outcomes after intracerebral haemorrhage (stroke severity and post-stroke mortality). Solid lines indicate the relationships between covariates, and the dotted line indicate the causal effect of physical activity.

For peer review only

**Table 1.** Registers and variables included in the study.

| | |
|---|---|
| **The Väststroke register** | |
| Age | Mean-centred. |
| Sex | Female/Male. |
| Physical activity assessment (SGPALS) | Mostly sedentary (Level 1); Light physical activity such as walking ≥ 4 hours/week (Level 2); Moderate physical activity such as running ≥ 2 hours/week (Level 3); Hard physical training for competition sports several times per week (Level 4). |
| Stroke severity assessment (NIHSS) | Very mild (NIHSS 0-1), mild (NIHSS 2-7), moderate (NIHSS 8-15), and severe (NIHSS ≥16) stroke. |
| Acute systolic blood pressure | Dichotomized based on median value. |
| Acute diastolic blood pressure | Dichotomized based on median value. |
| **The Riksstroke register** | |
| Prior Stroke | Yes/No. |
| Prior transient ischaemic attack | Yes/No. |
| Atrial fibrillation | Yes/No. |
| Diabetes | Yes/No. |
| Regular smoking the year before stroke | Yes/No. |
| Antihypertensive treatment | Yes/No. |
| Statin treatment | Yes/No. |
| Antiplatelet treatment | Yes/No. |
| Anticoagulant treatment | Yes/No. |
| **The LISA register** | |
| Country of birth | Born in Sweden/Born outside of Sweden. |
| Income | Ordered in quantiles (Q1 to Q5). |
| Educational level | Primary school (<10 years), secondary school (10-12 years), and postsecondary or university education (>12 years). |
| **The NPR** | |

For peer review only

| | |
|---|---|
| Charlson comorbidity index: Myocardial infarction, Heart failure, Peripheral vascular disease, Dementia, Pulmonary disease, Connective tissue disease, Peptic ulcer disease Liver disease, Diabetes, Hemiplegia or paraplegia, Renal disease, Cancer, HIV/AIDS | Low (0 points), moderate (1-2 points), high (3-4 points), and very high (≥5 points) risk. ICD-10 codes: I21, I22, I25.2, I42, I11.0, I13.0, I13.2 I50, I25.5, I65, I70-I74, I77, I73.9, I79.0, R02, F00-F03, F05.1, J40-J47, J60-J67, M05.0-M05.3, M05.8-M06.0, M06.3, M06.9, M32, M33.2, M34, M35.3, K25-K28, K70.2, K70.3, K71.7, K73, k74.0, K74.2-K74.6, K72.9, K76.6, K76.7, K72.1, E10.1, E10.5, E10.9, E11.1, E11.5, E11.9, E13.1, E13.5, E13.9, E14.1, E14.5, E14.9, E10.2-E10.4, E11.2-E11.4, E13.2-E13.4, E14.2-E14.4, G80.0, G80.2, G81, G82, N01-N08, N11, N14-N19, N25, C00-C76, C90, C97, C91-C95, C81-C86, C88, C77-C80, B20-B24 |
| Depression | Yes/No. ICD-10 codes: F32-F33 |
| Psychotic diseases | Yes/No. ICD-10 codes: F20-F29 |
| Alcohol related disorders | Yes/No. ICD-10 codes: F10, K70.1-K70.4, K70.9, K74.0 |
| Drug abuse | Yes/No. ICD-10 codes: F11, T40.0-T40.4, F12, T40.7, F14, T40.5, F15, T43.6, F16, T40.6, F19 |
| **The Swedish cause of death register** | **ICD-10 code** |
| All-cause mortality | - |
| Cerebrovascular mortality | ICD-10 codes: I60-I69 |

For peer review only

**Table 2.** Frequencies of stroke severity using the National Institutes of Health Stroke Scale (NIHSS), in physically inactive and active patients with stroke (Reinholdsson et al. 2018).[6]

| | No physical activity $\pi_C$ | Physical activity $\pi_E$ | Cumulative Odds ratio |
|---|---|---|---|
| Mild stroke (NIHSS 0-5) | 73.6% | 86.5% | 0.44 |
| Moderate stroke (NIHSS 6-14) | 20.4% | 10.8% | 0.43 |

| | | | |
|---|---|---|---|
| Severe stroke (NIHSS >14) | 6.0% | 2.7% | * |

For peer review only

**Contributorship statement**

A.V has taken part in the planning and design of this study. A.V will collect the data, take part in data analysis, and write the manuscript. D.B has taken part in the design of this study. D.B will take part in the data analysis and revise the manuscript. K.S.S has led the planning and design of this study. K.S.S will assist in the interpretation of data and revise the manuscript.

**Competing interests**

The authors report no conflict of interest

**Funding**

The study was funded by the Swedish Research Council (VR2017-00946), the Swedish Heart and Lung Foundation, the Swedish Brain Foundation, the Swedish Stroke Association, Promobilia, and the Swedish state under an agreement between the Swedish government and the county councils: the ALF agreement (ALFGBG-71980).

For peer review only

For peer review only

## References

1. Labrecque JA, Swanson SA. Target trial emulation: teaching epidemiology and beyond. *Eur J Epidemiol* 2017; **32**(6): 473-5.
2. Thomas L, Peterson ED. The value of statistical analysis plans in observational research: defining high-quality research from the start. *Jama* 2012; **308**(8): 773-4.
3. Deplanque D, Masse I, Lefebvre C, Libersa C, Leys D, Bordet R. Prior TIA, lipid-lowering drug use, and physical activity decrease ischemic stroke severity. *Neurology* 2006; **67**(8): 1403-10.
4. Deplanque D, Masse I, Libersa C, Leys D, Bordet R. Previous leisure-time physical activity dose dependently decreases ischemic stroke severity. *Stroke Research and Treatment* 2012; **2012**: 614925.
5. Krarup LH, Truelsen T, Gluud C, et al. Prestroke physical activity is associated with severity and long-term outcome from first-ever stroke. *Neurology* 2008; **71**(17): 1313-8.
6. Reinholdsson M, Palstam A, Sunnerhagen KS. Prestroke physical activity could influence acute stroke severity (part of PAPSIGOT). *Neurology* 2018; **91**(16): e1461-e7.
7. Ricciardi AC, Lopez-Cancio E, Perez de la Ossa N, et al. Prestroke physical activity is associated with good functional outcome and arterial recanalization after stroke due to a large vessel occlusion. *Cerebrovascular Diseases* 2014; **37**(4): 304-11.

8. Wen CP, Liu CH, Jeng JS, et al. Pre-stroke physical activity is associated with fewer post-stroke complications, lower mortality and a better long-term outcome. *European Journal of Neurology* 2017; **24**(12): 1525-31.
9. Mediano MFF, Mok Y, Coresh J, et al. Prestroke Physical Activity and Adverse Health Outcomes After Stroke in the Atherosclerosis Risk in Communities Study. *Stroke* 2021: Strokeaha120032695.
10. Bell CL, LaCroix A, Masaki K, et al. Prestroke factors associated with poststroke mortality and recovery in older women in the Women's Health Initiative. *Journal of the American Geriatrics Society* 2013; **61**(8): 1324-30.
11. Morovatdar N, Di Napoli M, Stranges S, et al. Regular physical activity postpones age of occurrence of first-ever stroke and improves long-term outcomes. *Neurol Sci* 2020.
12. Li HX, Wang PS, Tian GL, Xu WL, Liu D, Wu J. A seven-year follow-up analysis on prognosis factors of stroke patients. *Chung Hua Liu Hsing Ping Hsueh Tsa Chih* 2005; **26**(9): 716-9.
13. Stroud N, Mazwi TM, Case LD, et al. Prestroke physical activity and early functional status after stroke. *J Neurol Neurosurg Psychiatry* 2009; **80**(9): 1019-22.
14. Ursin MH, Ihle-Hansen H, Fure B, Tveit A, Bergland A. Effects of premorbid physical activity on stroke severity and post-stroke functioning. *Journal of Rehabilitation Medicine* 2015; **47**(7): 612-7.
15. Decourcelle A, Moulin S, Sibon I, et al. Influence of previous physical activity on the outcome of patients treated by thrombolytic therapy for stroke. *Journal of Neurology* 2015; **262**(11): 2513-9.
16. Redfors P, Isaksen D, Lappas G, et al. Living alone predicts mortality in patients with ischemic stroke before 70 years of age: a long-term prospective follow-up study. *BMC Neurology* 2016; **16**: 80.
17. Gamble C, Krishan A, Stocken D, et al. Guidelines for the Content of Statistical Analysis Plans in Clinical Trials. *Jama* 2017; **318**(23): 2337-43.
18. Saltin B, Grimby G. Physiological analysis of middle-aged and old former athletes. Comparison with still active athletes of the same ages. *Circulation* 1968; **38**(6): 1104-15.
19. Grimby G, Börjesson M, Jonsdottir IH, Schnohr P, Thelle DS, Saltin B. The "Saltin-Grimby Physical Activity Level Scale" and its application to health research. *Scand J Med Sci Sports* 2015; **25 Suppl 4**: 119-25.
20. Muir KW, Weir CJ, Murray GD, Povey C, Lees KR. Comparison of neurological scales and scoring systems for acute stroke prognosis. *Stroke* 1996; **27**(10): 1817-20.
21. Charlson ME, Pompei P, Ales KL, MacKenzie CR. A new method of classifying prognostic comorbidity in longitudinal studies: development and validation. *J Chronic Dis* 1987; **40**(5): 373-83.
22. Whitehead J. Sample size calculations for ordered categorical data. *Stat Med* 1993; **12**(24): 2257-71.
23. Rubin DB. Statistical Matching Using File Concatenation With Adjusted Weights and Multiple Imputations. *Journal of Business & Economic Statistics* 1986; **4**(1): 87-94.
24. van Buuren S, Groothuis-Oudshoorn K. mice: Multivariate Imputation by Chained Equations in R. *2011* 2011; **45**(3): 67.
25. Vink G, Frank LE, Pannekoek J, Buuren S. Predictive mean matching imputation of semicontinuous variables. *Statistica Neerlandica* 2014; **68**(1): 61-90.
26. McCaffrey DF, Ridgeway G, Morral AR. Propensity score estimation with boosted regression for evaluating causal effects in observational studies. *Psychol Methods* 2004; **9**(4): 403-25.
27. Ho DE, Imai K, King G, Stuart EA. Matching as Nonparametric Preprocessing for Reducing Model Dependence in Parametric Causal Inference. *Political Analysis* 2007; **15**(3): 199-236.
28. Christensen R. Cumulative Link Models for Ordinal Regression with the R Package ordinal. 2018; 2018.
29. Liu D, Zhang H. Residuals and Diagnostics for Ordinal Regression Models: A Surrogate Approach. *Journal of the American Statistical Association* 2018; **113**: 1-10.

For peer review only

30. Cox DR. Regression Models and Life-Tables. *Journal of the Royal Statistical Society: Series B (Methodological)* 1972; **34**(2): 187-202.
31. Borgan Ø. Modeling Survival Data: Extending the Cox Model. Terry M. Therneau and Patricia M. Grambsch, Springer-Verlag, New York, 2000. ISBN 0-387-98784-3. *Statistics in Medicine* 2001; **20**(13): 2053-4.
32. Xue Y, Schifano ED. Diagnostics for the Cox model. *CSAM* 2017; **24**(6): 583-604.
33. Bagg S, Pombo AP, Hopman W. Effect of age on functional outcomes after stroke rehabilitation. *Stroke* 2002; **33**(1): 179-85.
34. Sacco S, Marini C, Toni D, Olivieri L, Carolei A. Incidence and 10-year survival of intracerebral hemorrhage in a population-based registry. *Stroke* 2009; **40**(2): 394-9.
35. King AC, King DK. Physical Activity for an Aging Population. *Public Health Reviews* 2010; **32**(2): 401-26.
36. Phan HT, Reeves MJ, Blizzard CL, et al. Sex Differences in Severity of Stroke in the INSTRUCT Study: a Meta-Analysis of Individual Participant Data. *J Am Heart Assoc* 2019; **8**(1): e010235.
37. Lindmark A, Glader E-L, Asplund K, Norrving B, Eriksson M. Socioeconomic Disparities in Stroke Case Fatality – Observations from Riks-Stroke, the Swedish Stroke Register. *International Journal of Stroke* 2014; **9**(4): 429-36.
38. Lindmark A, Norrving B, Eriksson M. Socioeconomic status and survival after stroke – using mediation and sensitivity analyses to assess the effect of stroke severity and unmeasured confounding. *BMC Public Health* 2020; **20**(1): 554.
39. O'Donoghue G, Kennedy A, Puggina A, et al. Socio-economic determinants of physical activity across the life course: A "DEterminants of DIet and Physical ACtivity" (DEDIPAC) umbrella literature review. *PLoS One* 2018; **13**(1): e0190737.
40. Miyares LC, Falcone GJ, Leasure A, et al. Race/ethnicity influences outcomes in young adults with supratentorial intracerebral hemorrhage. *Neurology* 2020; **94**(12): e1271-e80.
41. Edjoc RK, Reid RD, Sharma M, Fang J. The prognostic effect of cigarette smoking on stroke severity, disability, length of stay in hospital, and mortality in a cohort with cerebrovascular disease. *Journal of stroke and cerebrovascular diseases : the official journal of National Stroke Association* 2013; **22**(8): e446-54.
42. Weng WC, Huang WY, Chien YY, et al. The impact of smoking on the severity of acute ischemic stroke. *J Neurol Sci* 2011; **308**(1-2): 94-7.
43. Juvela S. Risk factors for impaired outcome after spontaneous intracerebral hemorrhage. *Arch Neurol* 1995; **52**(12): 1193-200.
44. Kaczynski AT, Manske SR, Mannell RC, Grewal K. Smoking and physical activity: a systematic review. *Am J Health Behav* 2008; **32**(1): 93-110.
45. Vancampfort D, De Hert M, Stubbs B, et al. A systematic review of physical activity correlates in alcohol use disorders. *Arch Psychiatr Nurs* 2015; **29**(4): 196-201.
46. Lin CY, Chien CC, Chen HA, et al. The impact of comorbidity on survival after hemorrhagic stroke among dialysis patients: a nationwide population-based study. *BMC Nephrol* 2014; **15**: 186.
47. Liu H, Wu X, Cao J, et al. Effect of Comorbidity Assessed by the Charlson Comorbidity Index on the Length of Stay and Mortality Among Immobile Hemorrhagic Stroke Patients Younger Than 50 Years. *Frontiers in neurology* 2020; **11**: 487.
48. Hoyer C, Schmidt HL, Kranaster L, Alonso A. Impact of psychiatric comorbidity on the severity, short-term functional outcome, and psychiatric complications after acute stroke. *Neuropsychiatr Dis Treat* 2019; **15**: 1823-31.
49. Camacho TC, Roberts RE, Lazarus NB, Kaplan GA, Cohen RD. Physical Activity and Depression: Evidence from the Alameda County Study. *American Journal of Epidemiology* 1991; **134**(2): 220-31.
50. Roshanaei-Moghaddam B, Katon WJ, Russo J. The longitudinal effects of depression on physical activity. *General Hospital Psychiatry* 2009; **31**(4): 306-15.

For peer review only

51. Stubbs B, Firth J, Berry A, et al. How much physical activity do people with schizophrenia engage in? A systematic review, comparative meta-analysis and meta-regression. *Schizophrenia Research* 2016; **176**(2): 431-40.
52. Passero S, Ciacci G, Ulivelli M. The influence of diabetes and hyperglycemia on clinical course after intracerebral hemorrhage. *Neurology* 2003; **61**(10): 1351-6.
53. Fogelholm R, Murros K, Rissanen A, Avikainen S. Admission blood glucose and short term survival in primary intracerebral haemorrhage: a population based study. *J Neurol Neurosurg Psychiatry* 2005; **76**(3): 349-53.
54. Kimura K, Iguchi Y, Inoue T, et al. Hyperglycemia independently increases the risk of early death in acute spontaneous intracerebral hemorrhage. *J Neurol Sci* 2007; **255**(1-2): 90-4.
55. Trovati M, Carta Q, Cavalot F, et al. Influence of physical training on blood glucose control, glucose tolerance, insulin secretion, and insulin action in non-insulin-dependent diabetic patients. *Diabetes Care* 1984; **7**(5): 416-20.
56. Horstmann S, Rizos T, Jenetzky E, Gumbinger C, Hacke W, Veltkamp R. Prevalence of atrial fibrillation in intracerebral hemorrhage. *European Journal of Neurology* 2014; **21**(4): 570-6.
57. Roquer J, Vivanco-Hidalgo RM, Prats-Sánchez LL, et al. Interaction of atrial fibrillation and antithrombotics on outcome in intracerebral hemorrhage. *Neurology* 2019; **93**(19): e1820-e9.
58. Nattel S. Physical activity and atrial fibrillation risk: it's complicated; and sex is critical. *European heart journal* 2020; **41**(15): 1487-9.
59. Flibotte JJ, Hagan N, O'Donnell J, Greenberg SM, Rosand J. Warfarin, hematoma expansion, and outcome of intracerebral hemorrhage. *Neurology* 2004; **63**(6): 1059-64.
60. Rosand J, Eckman MH, Knudsen KA, Singer DE, Greenberg SM. The effect of warfarin and intensity of anticoagulation on outcome of intracerebral hemorrhage. *Archives of internal medicine* 2004; **164**(8): 880-4.
61. Wilson D, Seiffge DJ, Traenka C, et al. Outcome of intracerebral hemorrhage associated with different oral anticoagulants. *Neurology* 2017; **88**(18): 1693-700.
62. Puddey IB. Low serum cholesterol and the risk of cerebral haemorrhage. *Atherosclerosis* 1996; **119**(1): 1-6.
63. Rodriguez-Luna D, Rubiera M, Ribo M, et al. Serum low-density lipoprotein cholesterol level predicts hematoma growth and clinical outcome after acute intracerebral hemorrhage. *Stroke* 2011; **42**(9): 2447-52.
64. Feng H, Wang X, Wang W, Zhao X. Lipid Levels and 3-Month Prognosis After Spontaneous Intracerebral Hemorrhage in Women. *Frontiers in neurology* 2021; **12**(963).
65. Winkler J, Shoup JP, Czap A, et al. Long-term improvement in outcome after intracerebral hemorrhage in patients treated with statins. *Journal of stroke and cerebrovascular diseases : the official journal of National Stroke Association* 2013; **22**(8): e541-5.
66. Siddiqui FM, Langefeld CD, Moomaw CJ, et al. Use of Statins and Outcomes in Intracerebral Hemorrhage Patients. *Stroke* 2017; **48**(8): 2098-104.
67. Durstine JL, Grandjean PW, Cox CA, Thompson PD. Lipids, lipoproteins, and exercise. *J Cardiopulm Rehabil* 2002; **22**(6): 385-98.

For peer review only

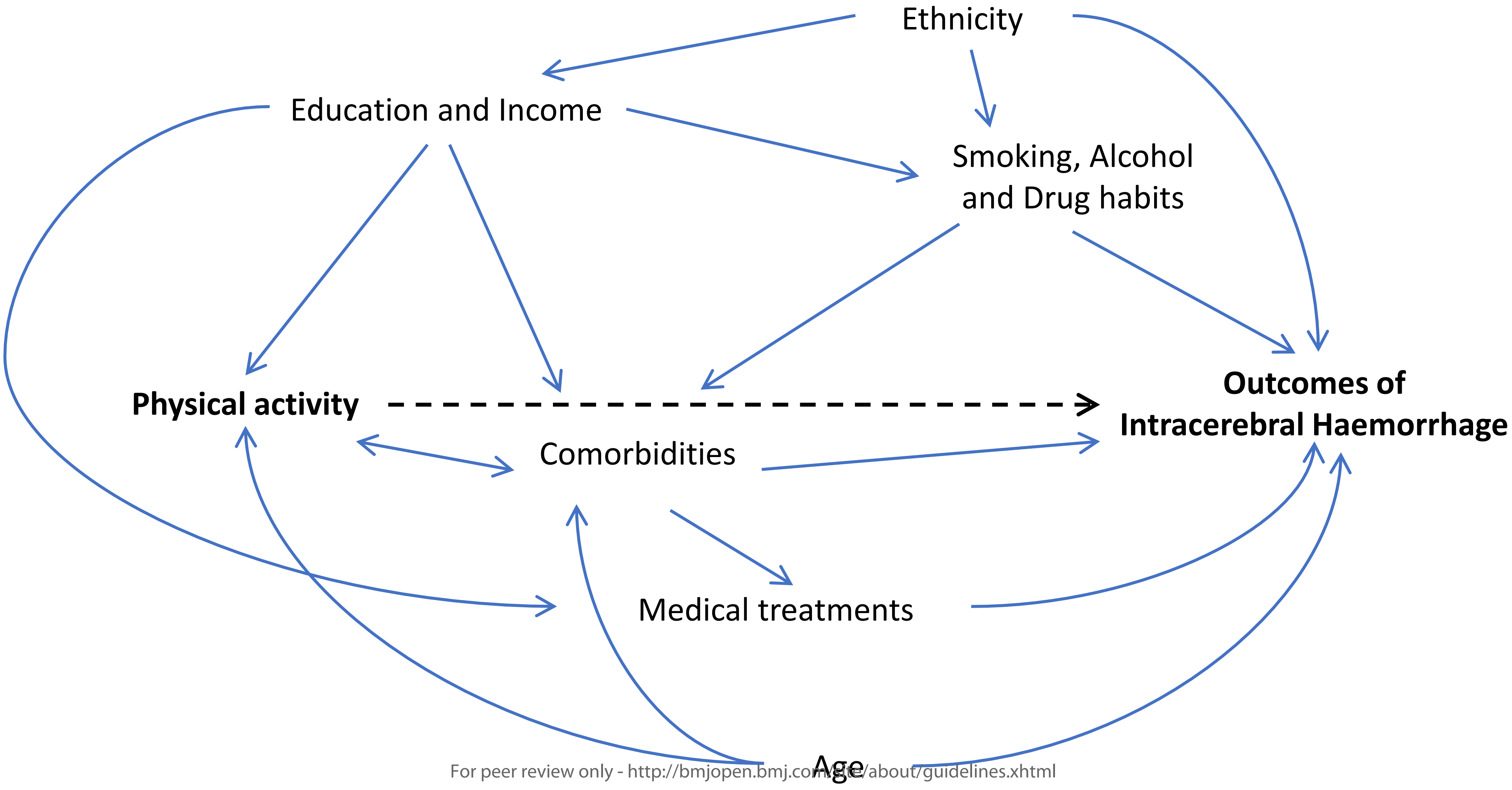


For peer review only - http://bmjopen.bmj.com/site/about/guidelines.xhtml
